# An in depth investigation on device-to-device communication in heterogeneous networks: opportunities and challenges

**DOI:** 10.1016/j.mex.2025.103777

**Published:** 2025-12-17

**Authors:** Amjad Ali, Aqsa Zehra, Muhammad Nafees, Muhammad Awais Amin

**Affiliations:** a Karachi Institute of Economic and Technology, Pakistan; b Data Science Consultant Datamatics Technologies, Gulburg Greens, Islamabad, Pakistan

**Keywords:** UAVs D2D transmission, Cellular network, 5G environment, Public safety scenarios, Military domain, Social networks, Ad-hoc networks, IoT domain

## Abstract

Accurate data from appropriate areas and in rural areas collect reliably and quickly with the help of UAVs. UAVs are used to gather any type of information either from land, sea, or air. The communication between UAV to UAV and UAV to the ground requires some linkage or a path for transmission. Device-to-device transmission is used to offload traffic in a congestion network. However, a D2D transmission is based on multiple networks. All these networks are collectively discussed in this paper included; cellular, 5G, military, ad-hoc, IoT, Social, and public safety scenarios. A variety of advantages in terms of reliability, latency, spectrum efficiency, energy efficiency, interference mitigation, high altitude, power optimization, autonomous relaying, increased throughput, high data rates, privacy, security, mode selection, FANET, MANET, VANET, coverage extension, and covert communication is given in this paper. Eventually, in this paper, we provide all the opportunities and challenges in D2D transmission over contrasting network domains.

## Review Highlights

• The communication between UAV to UAV and UAV to the ground requires some linkage or a path for transmission. Device-to-device transmission is used to offload traffic in a congestion network.
• D2D transmission is based on multiple networks. All these networks are collectively discussed in this paper included; cellular, 5G, military, ad-hoc, IoT, Social, and public safety scenarios.
• A variety of advantages in terms of reliability, latency, spectrum efficiency, energy efficiency, interference mitigation, high altitude, power optimization, autonomous relaying, increased throughput, high data rates, privacy, security, mode selection, FANET, MANET, VANET, coverage extension and covert communication are discussed in this paper.

## Graphical abstract

**Figure:**
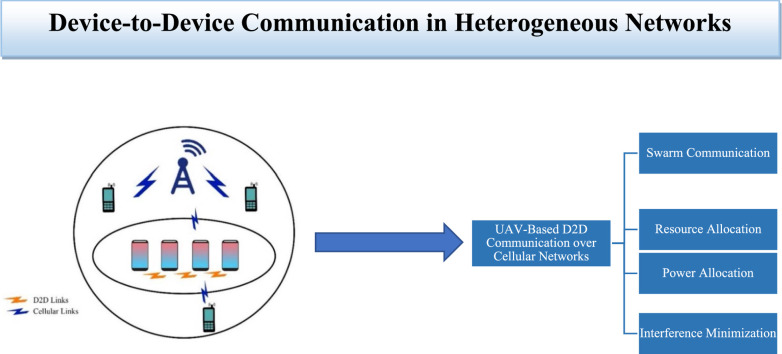

Specifications table

**Table:** 

**Subject area**	Computer Science
**More specific subject area**	*Device-to-Device UAV Communication in Heterogeneous Networks*
**Name of the reviewed methodology**	*Research Questions and Survey Research*
**Keywords**	*UAVs D2D transmission; Cellular network; 5G environment; Public safety scenarios; Military domain; Social networks; Ad-hoc networks; IoT domain;*
**Resource availability**	*[Not applicable]*
**Review question**	***RQ 1****:How does device-to-device (D2D) communication perform across different types of wireless networks (e.g., 4G, 5G, Wi-Fi, LoRaWAN)?* ***RQ 2****:What are the primary security and privacy challenges faced in D2D transmission over heterogeneous networks?* ***RQ 3****:What are the solutions of security and privacy in D2D transmission over heterogeneous networks?*

## Background

D2D transmission in UAVs gets popular in **military networks**. Most of the research was carried out in the military domain using D2D links among which jamming and selection of mode for convert communication in the network were done [3]. Many proposed algorithms were designed in the military zone for future applications which would be more sensitive. The protocols were used in D2D links to support the security of transmission using formal authentication and verification [14]. Block chain technology become popular for trusted and secured transmission beyond **5G** to make the D2D link among UAVs secured and saved [[18], [26]].An autonomous relaying routing algorithm when using social networks was presented, for that purpose optimization technique was performed [32]. Multiple input multiple output (MIMO) relaying for the **safety of the public** in disastrous scenarios [33]. 5G was introduced in military communication and focused on security challenges and services by the 3Gpp group [4]. A concept of a swarm in UAVs using D2D links was also introduced over **cellular networks**. A reliable performance was carried out in the swarm [11]. The concept of Flying Adhoc Network (FANET) was presented which enables wireless technologies, applications, and challenges [24]. In a 5G network environment, the challenges and issues were proposed [39]. UAVs using D2D links were also used in IoT in a 5G environment, and to make a network secure lightweight cryptography was presented [22]. A secure authentication channel was designed with ultra-reliable low latency. As the IoT devices were constrained in resources so the issues for security become more crucial. D2D transmissions were also seen in **social networks**, offloading traffic was carried out from the congestion cellular networks [31]. D2D transmission over cellular networks was presented with a distributed routing algorithm in a 5G environment which enhanced the coverage and broadcast in a wide range [2,45]. In the **IoT domain,** a wireless network for the safety of the public uses adaptive-learning analysis and makes the system energy-efficient [25].The **ad-Hoc cellular network** deployed for VANET (Vehicular Adhoc Network) was lightweight LTE networks [[7], [43]]. In Het-IoT UAV guided in emergency transmission using distributed SIC Free-Non-Orthogonal Multiple Access (DSF-NOMA) technique [21]. In VANET safety was taken in D2D transmission which was used in cluster form [23].A clustering schemes used to expand cellular coverage for public safety [30]. For IoT-based coverage in disastrous areas was done by using multi-hop D2D links between UAVs [34]. D2D transmission between UAVs in the social domain is done by optimizing power and convex optimization [38]. Relay selection for 5G cellular networks in D2D transmission to increase spectrum efficiency and expand coverage [13]. In D2D transmission over cellular networks, the interference was minimized by decreasing the sum rate [20].Resilient smart cities for the recovery of networks in disastrous areas were developed by empowering drones using small cellular networks [1,44]. In a 5G environment, the interference in D2D transmission and the challenges in that environment were cured over cellular networks and the interference was minimized in 5G [19,46].

## Method details

For the safe and secure transmission using D2D links in 5G, some key protocols were designed to enhance the coverage of the system [28]. A delay-tolerant network (DTN) scheme was proposed for ad-hoc based D2D transmission in cellular networks [37]. To extend the coverage in the scenario for public safety using D2D transmission and multi-hopping was done and developed 3GPP simulator to mitigate interference and minimize power consumption [16]. In wireless networks, D2D transmission was aware in the social networks. Cumulative Distribution Function (CDF) was designed to offload traffic from the network for optimizing D2D transmission [35]. Under a cellular network, a distributed resource allocation for D2D transmission was done. The scheme is proposed with limited overheads and mitigates interference. D2D pairs provided with high data rates [29,47]. D2D transmission over the cellular network based on power allocation for full-duplex relaying. Power optimization was done in a closed-form [10].

The UAV-based D2D transmission was performed under different network domains which were highlighted above. The domains included cellular network, 5G network, military network, social network, ad-hoc network, IoT based and in public safety scenarios. The most advanced algorithms were proposed during a period that was implemented and better performance was achieved promptly. My contribution is to collect the different and massive spread of UAV-based D2D transmission over different networks on one platform to provide effective and efficient results under one head. These seven different cases for D2D transmission are further divided into five other papers under each head which provide the best results. The challenges and opportunities in UAV based D2D transmission over each network is discussed in this paper to provide a comprehensive study for more clear understanding to work further and introduce new schemes which would never be done before or to upgrade the existing algorithms to safe time (time efficiency) and improve results. Device-to-device (D2D) communication and cognitive radio are combined to create cognitive D2D (cD2D) communication, which has many benefits, including better coverage, higher network throughput, and energy and spectrum efficiency are also discussed [40].

## UAV-based D2D communication across network domains

In the context of UAV-based Device-to-Device (D2D) communication, several network domains play a critical role in optimizing communication efficiency, reducing latency, and improving network resilience. These domains include Cellular Networks, 5G, IoT, Military, Ad-Hoc Networks, Social Networks, and Power Allocation and Interference Mitigation Strategies. To understand the integration of UAVs into these networks, it is essential to visualize how each domain interacts within the overall system. Fig. 1, Fig. 2, Fig. 3, Fig. 4, Fig. 5, Fig. 6, Fig. 7, Fig. 8, Fig. 9, Fig. 10, Fig. 11, Fig. 12, Fig. 13, Fig. 14, Fig. 15, Fig. 16, Fig. 17, Fig. 18, Fig. 19, Fig. 20, Fig. 21, Fig. 22, Fig. 23, Fig. 24, Fig. 25, Fig. 26, Fig. 27, Fig. 28, Fig. 29, Fig. 30, Fig. 31, Fig. 32, Fig. 33, Fig. 34, Fig. 35, Fig. 36, Fig. 37, Fig. 38, Fig. 39, Fig. 40, Fig. 41

**Fig. 1:**
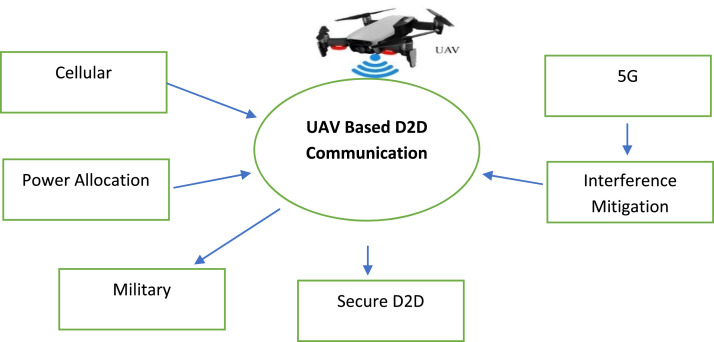
UAV-based D2D communication across network domains.

**Fig. 2:**
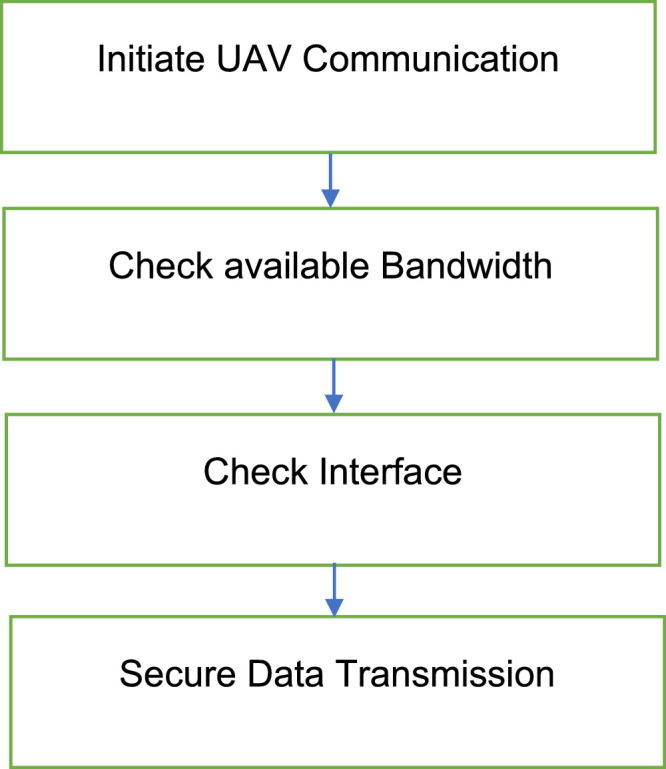
Network architecture flowchart for UAVs in 5G network.

**Fig. 3:**
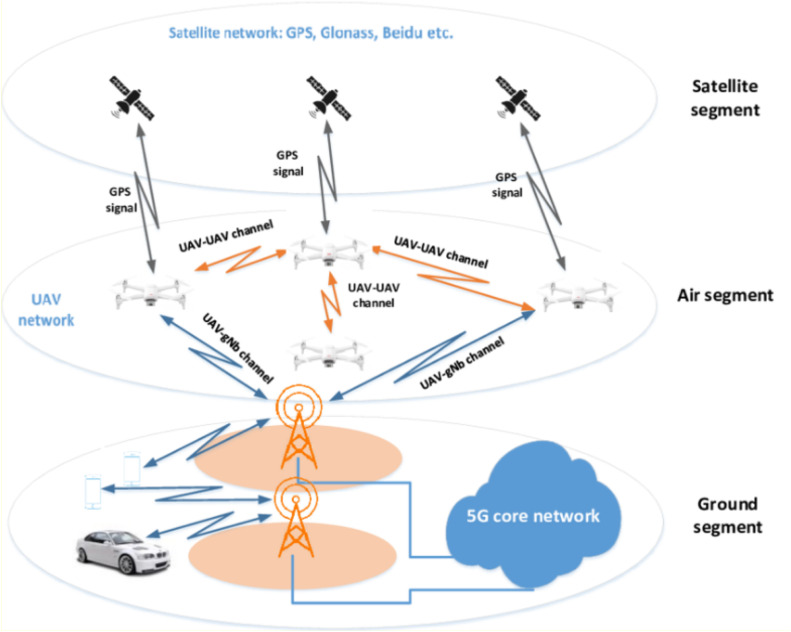
Network architecture for UAVs in 5G.

**Fig. 4:**
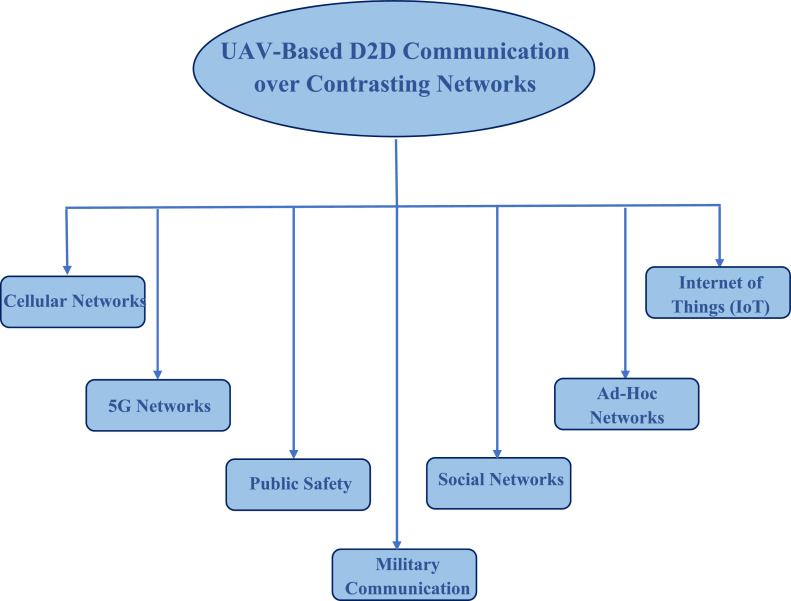
Research domains of this survey.

**Fig. 5:**
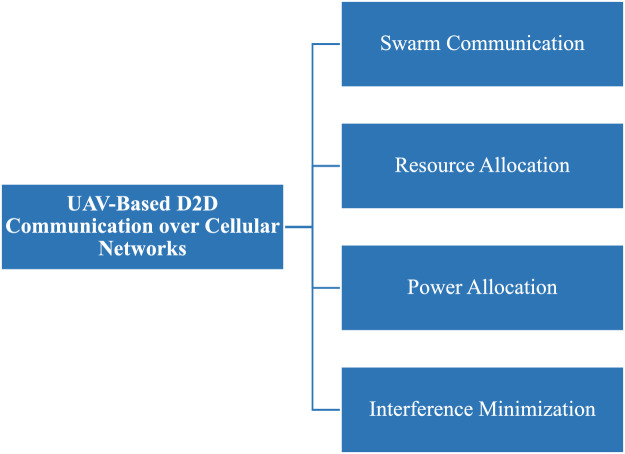
D2D Transmission over cellular network.

**Fig. 6:**
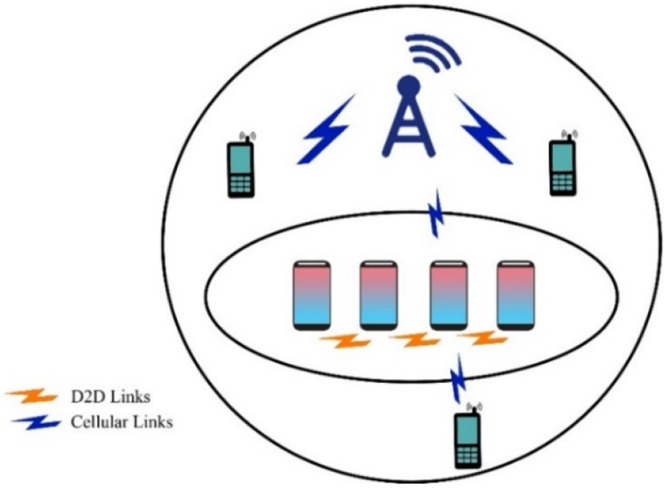
D2D Transmission over cellular network scenario.

**Fig. 7:**
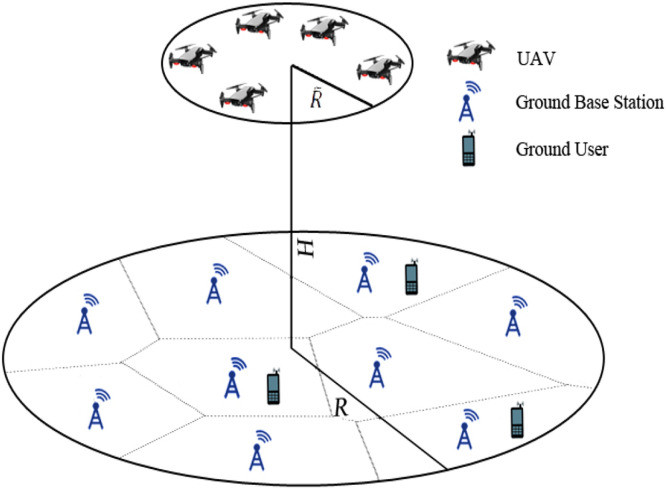
Cellular connected swarm of drones.

**Fig. 8:**
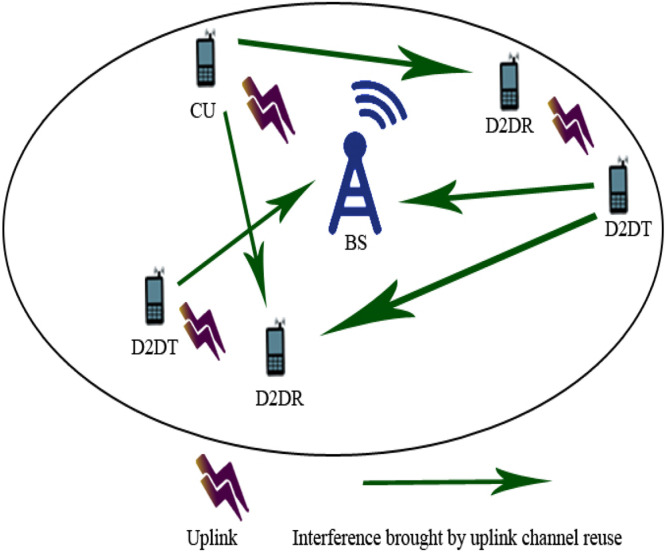
D2D communication underlying cellular networks when uplink channels are reused.

**Fig. 9:**
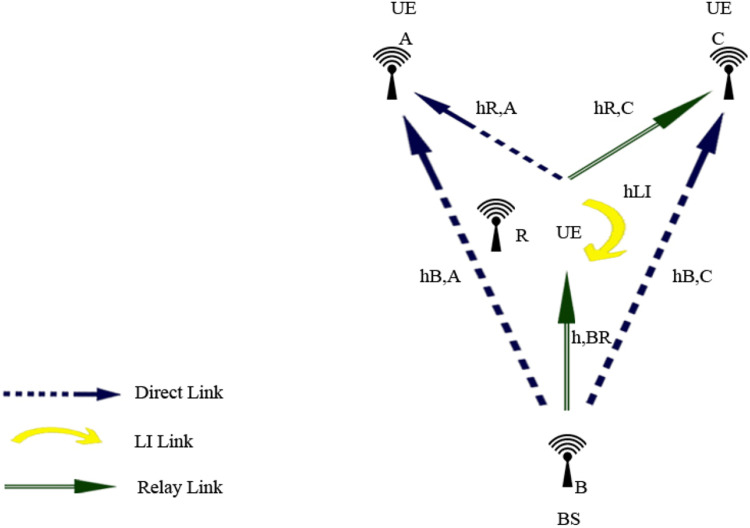
D2D Communication underlying cellular network model.

**Fig. 10:**
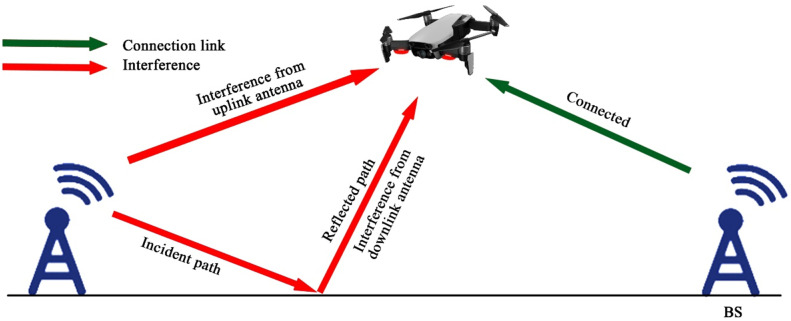
Interference from an uplink antenna and a reflected path in D2D transmission.

**Fig. 11:**
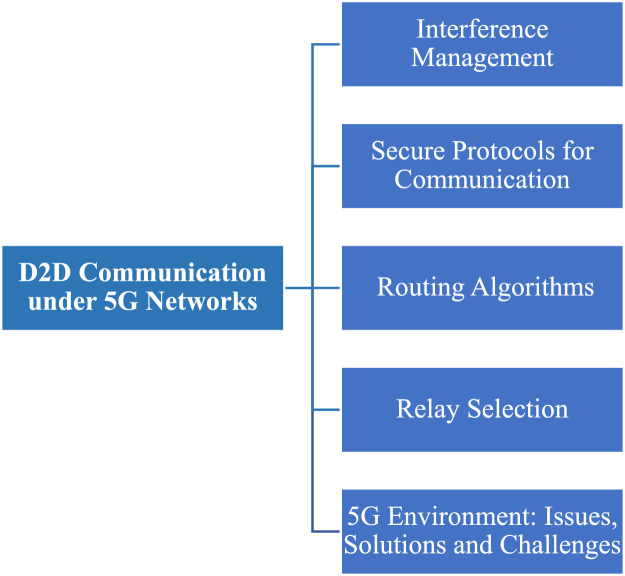
D2D transmission over 5G networks.

**Fig. 12:**
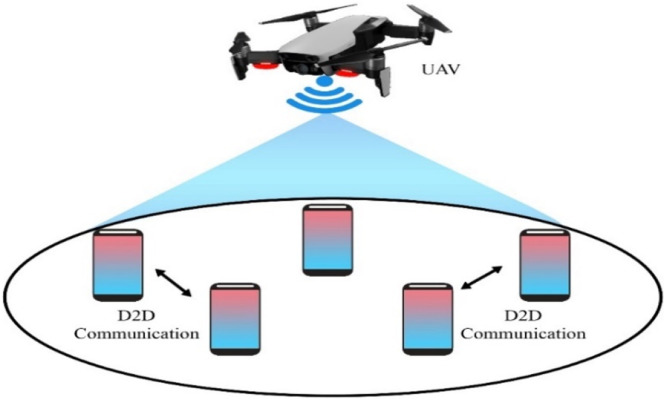
D2D transmission over 5G networks scenario.

**Fig. 13:**
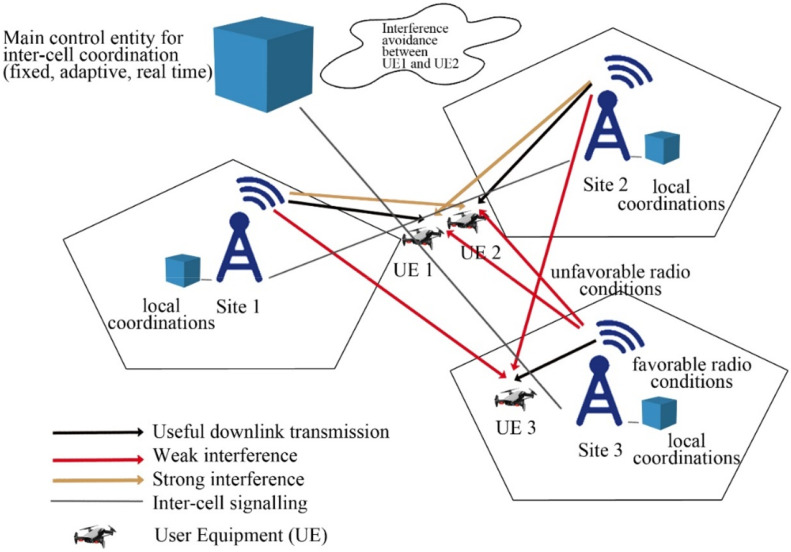
(a) D2D Intercell interference.

**Fig. 14:**
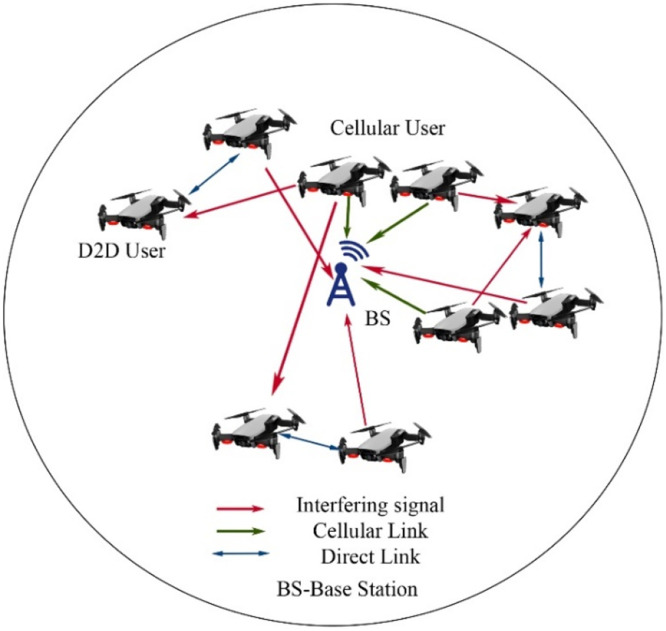
(b) D2D Intercell interference.

**Fig. 15:**
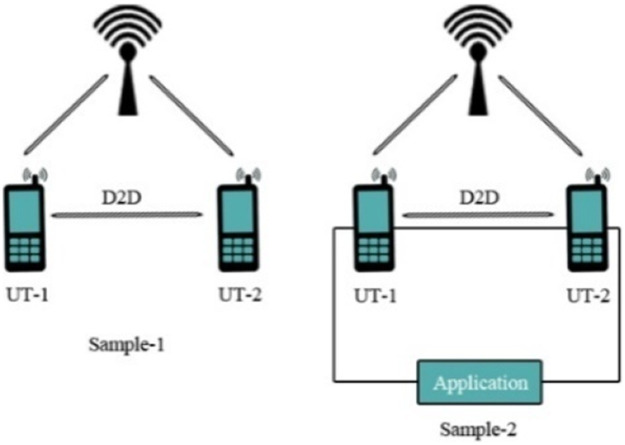
Secure D2D communication in 5G network.

**Fig. 16:**
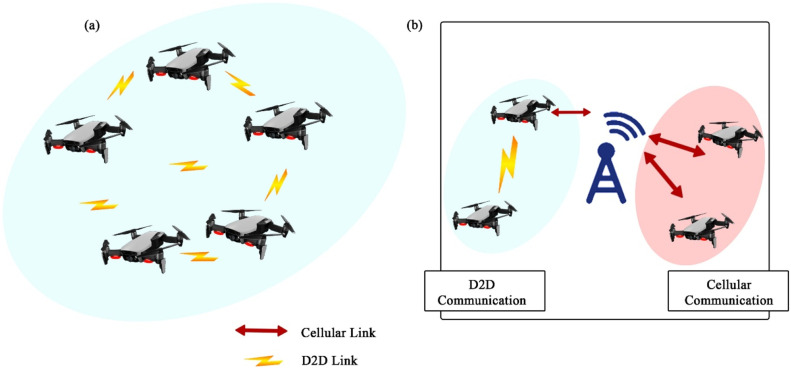
(a) Without infrastructure (standalone D2D) 15 (b) with infrastructure (network-assisted D2D).

**Fig. 17:**
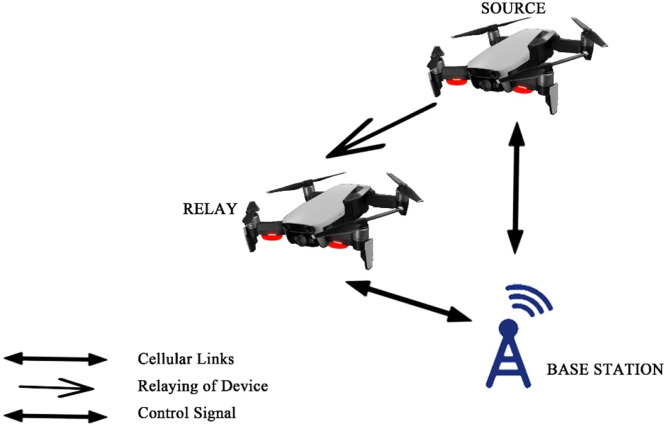
Relaying gadget with a command from Base Station.

**Fig. 18:**
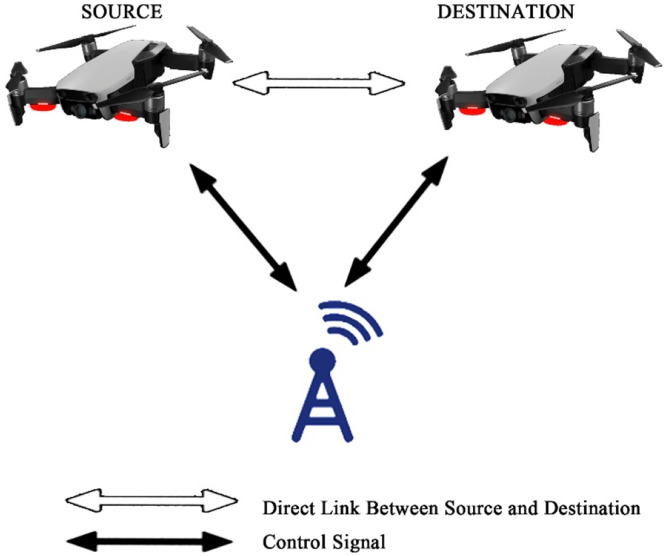
Direct communication between gadgets with control of Base Station.

**Fig. 19:**
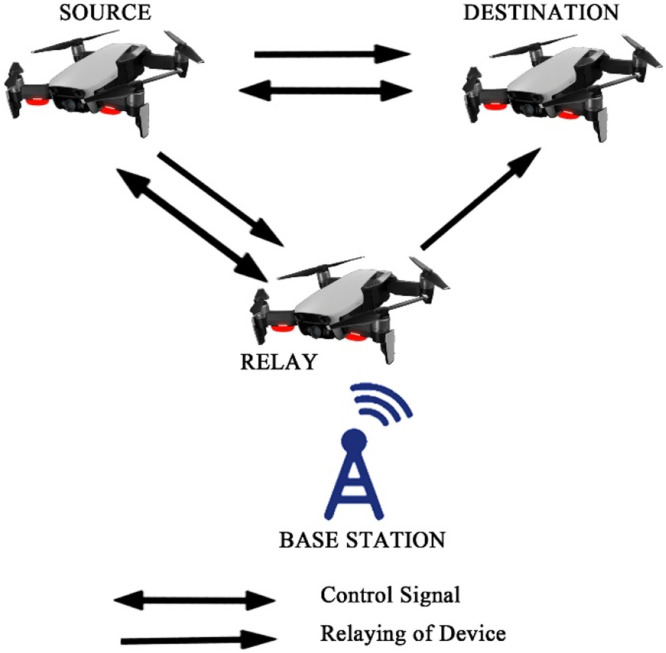
Device relaying fully controlled with gadget.

**Fig. 20:**
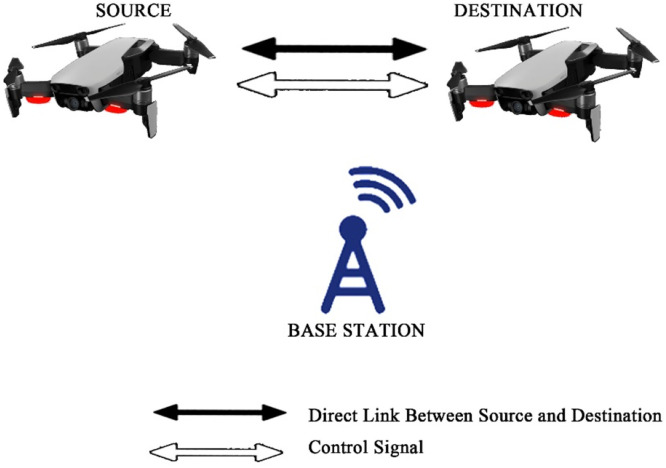
Direct communication between gadgets.

**Fig. 21:**
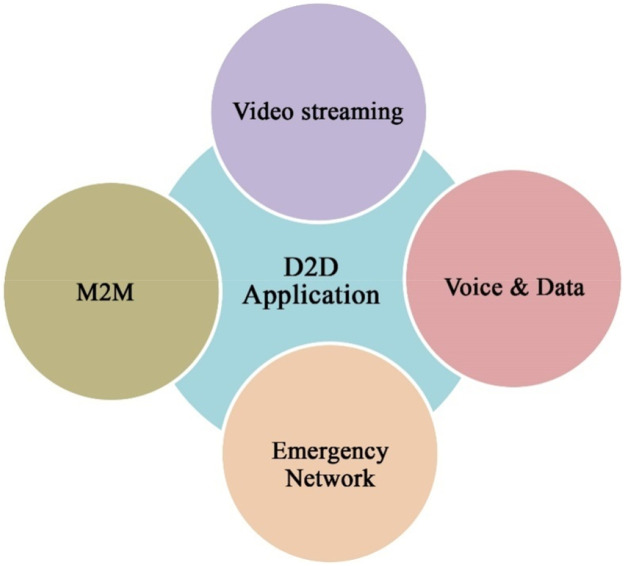
Challenges of D2D transmission in 5G environment.

**Fig. 22:**
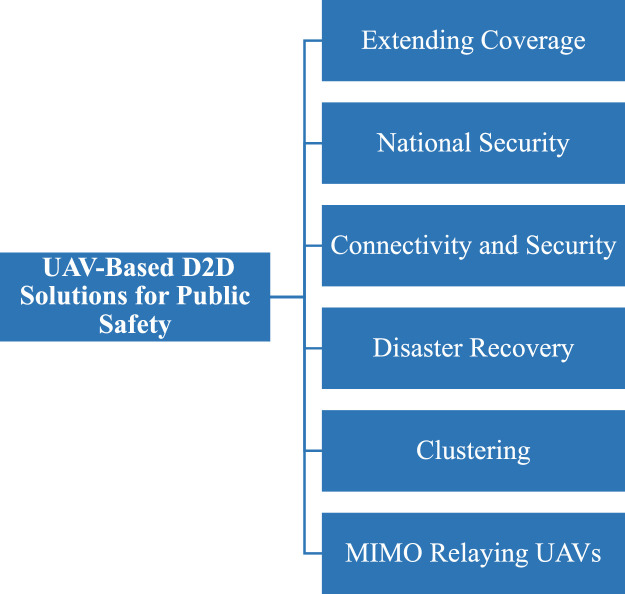
UAV-based D2D solutions for public safety.

**Fig. 23:**
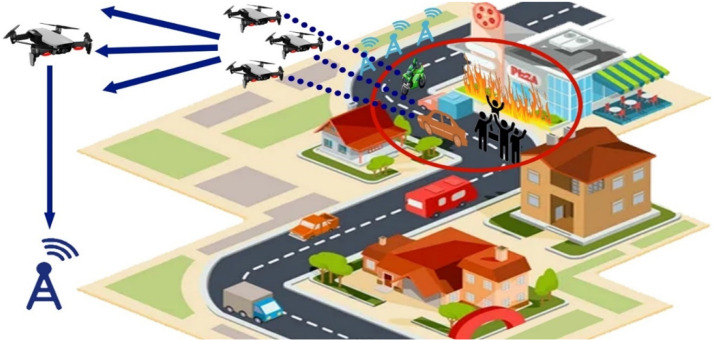
D2D transmission in public safety scenarios.

**Fig. 24:**
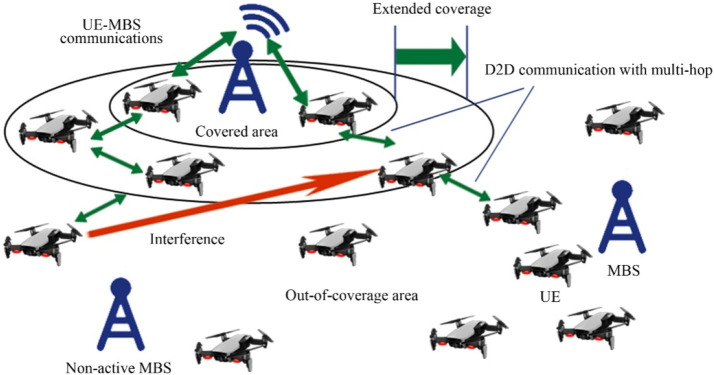
Extended coverage using D2D and multi-hop in public safety communication scenario with partial coverage.

**Fig. 25:**
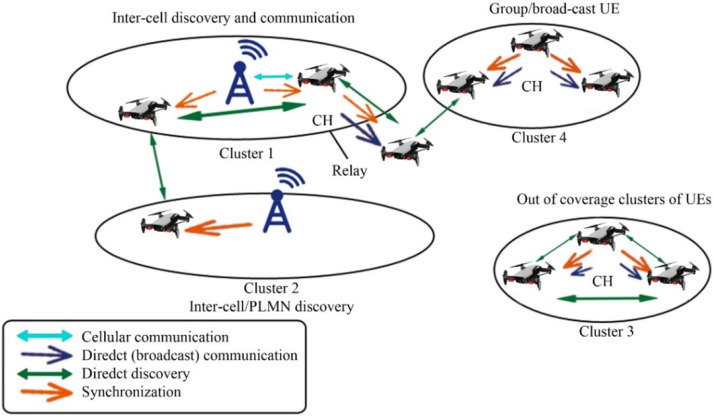
Synchronization provided in 4 clusters supporting scenario.

**Fig. 26:**
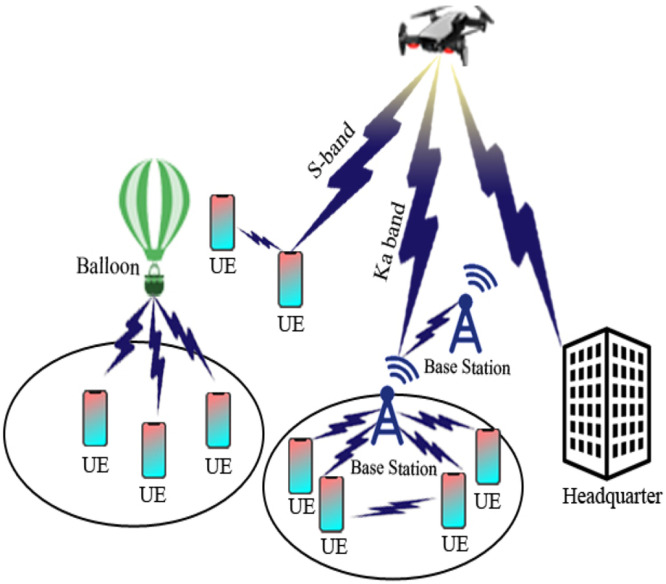
scenario of balloon project for public safety communication.

**Fig. 27:**
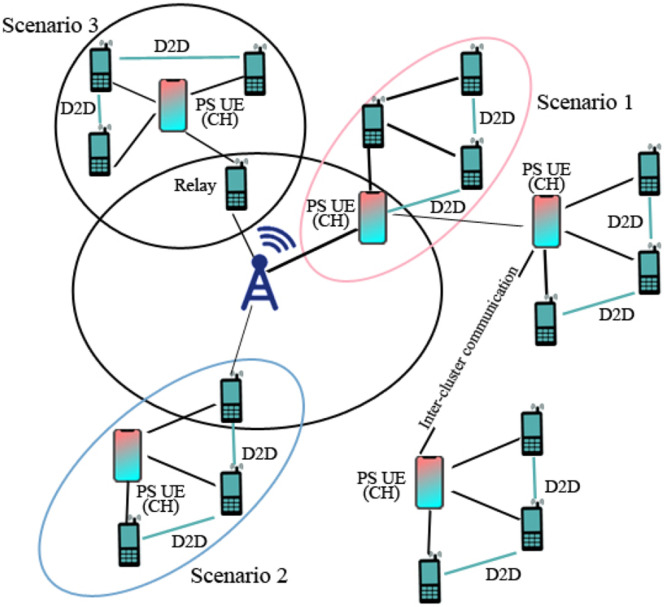
Network model for different scenarios.

**Fig. 28:**
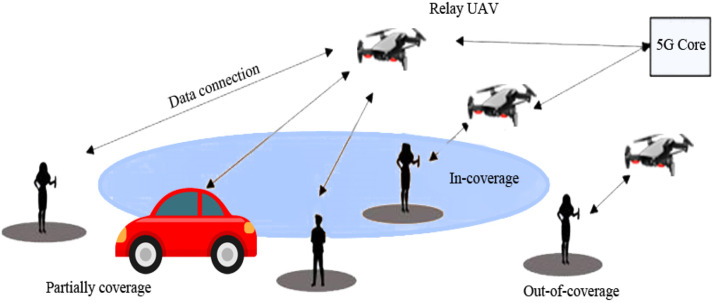
Public safety scenario available D2D communication using UAVs.

**Fig. 29:**
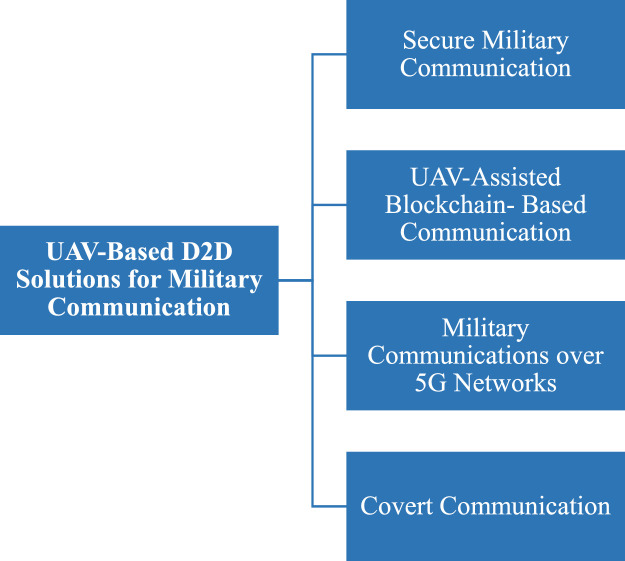
UAV-based D2D solutions for military communication solutions.

**Fig. 30:**
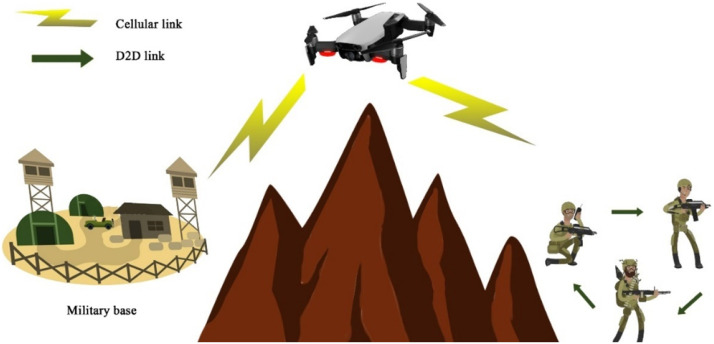
D2D transmission over military networks.

**Fig. 31:**
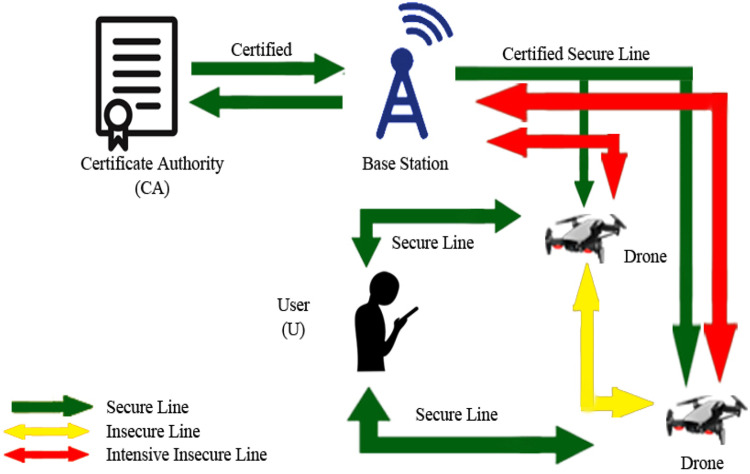
Execution flow of the proposed protocol.

**Fig. 32:**
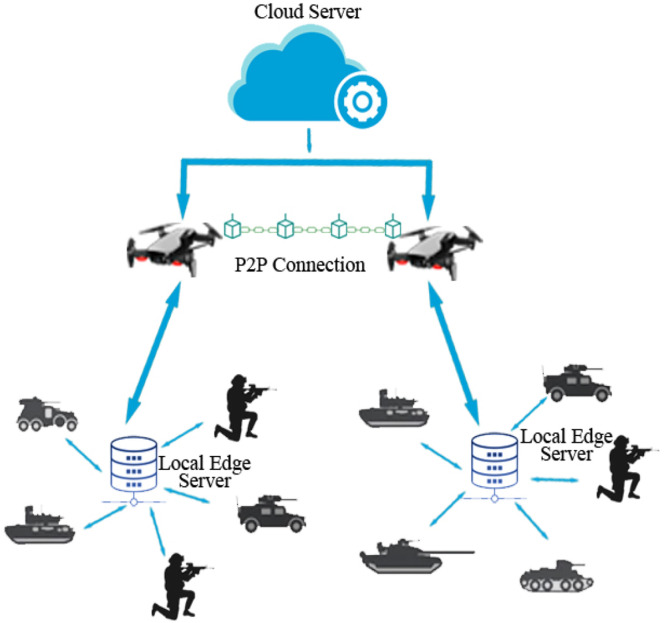
Blockchain-based communication in military network.

**Fig. 33:**
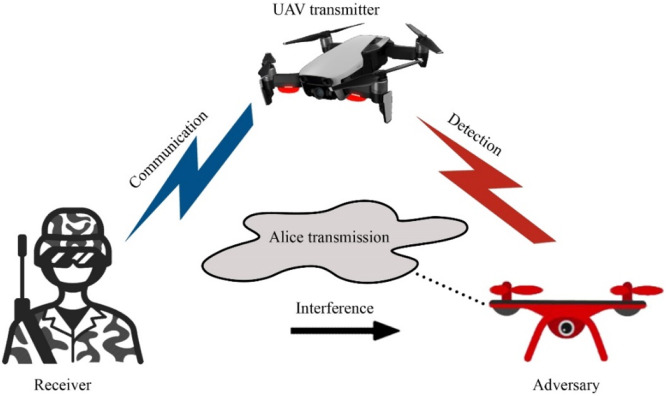
Covert Communication.

**Fig. 34:**
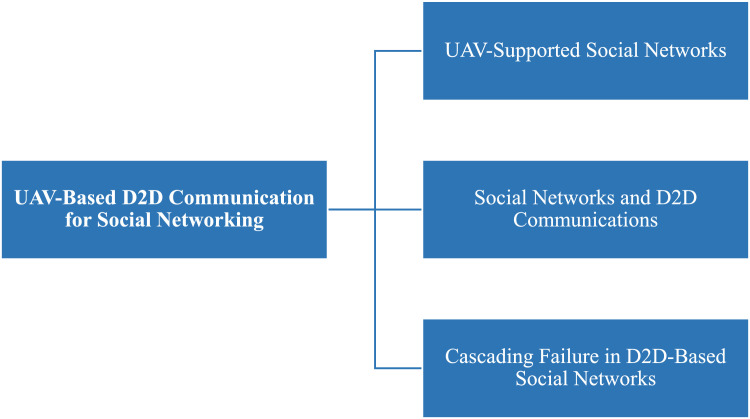
UAV-based D2D communication for social networking solutions.

**Fig. 35:**
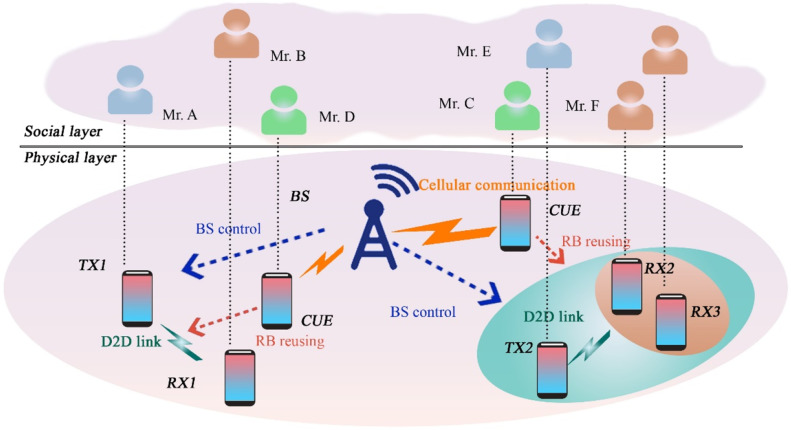
D2D transmission over social network.

**Fig. 36:**
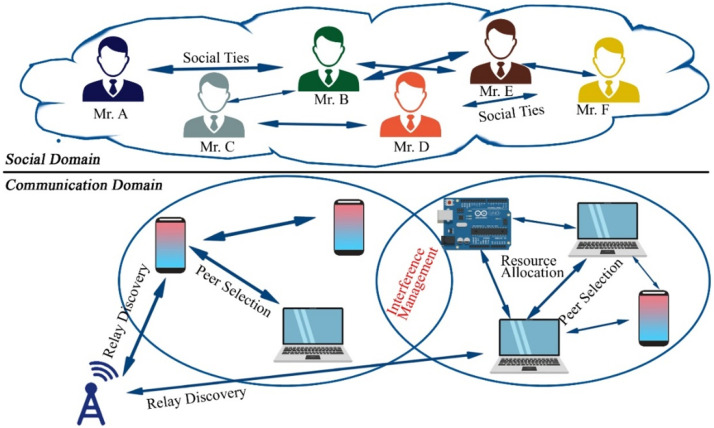
System model.

**Fig. 37:**
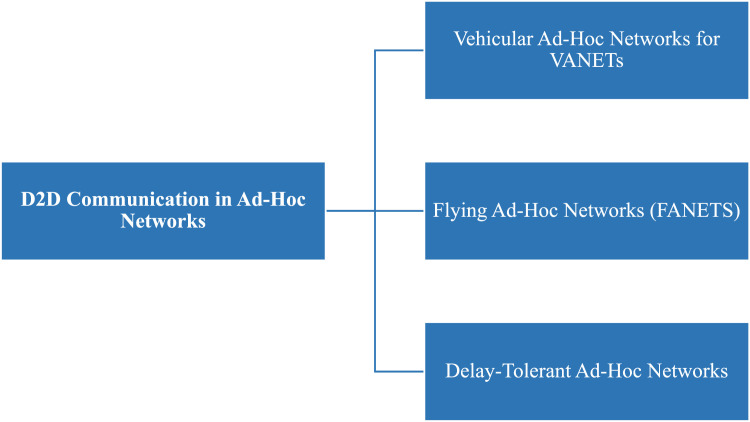
D2D communication in Ad-Hoc networks solutions.

**Fig. 38:**
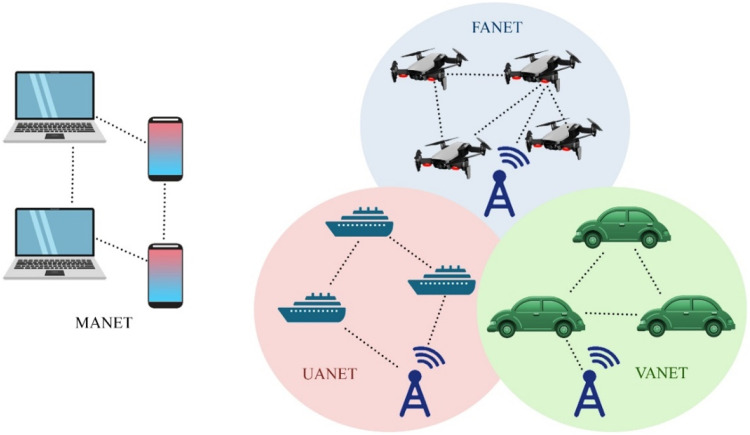
D2D transmission over Ad-Hoc networks.

**Fig. 39:**
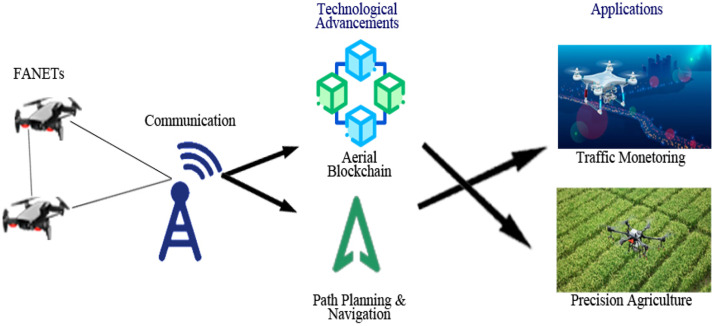
Scope of communication with technological advancements for various applications in FANETS.

**Fig. 40:**
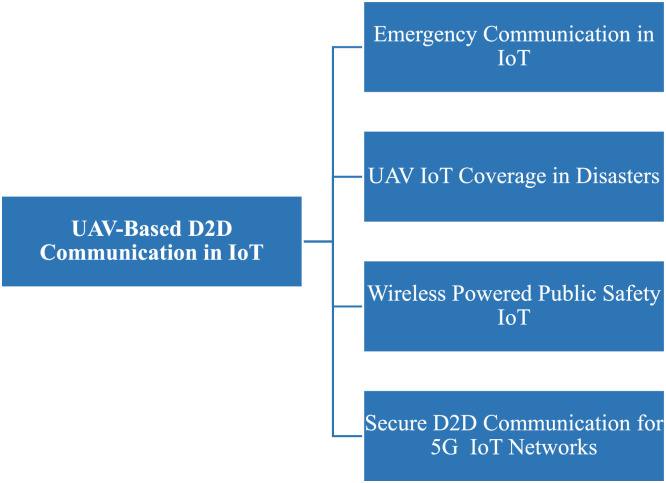
D2D transmission in IoT based networks.

**Fig. 41:**
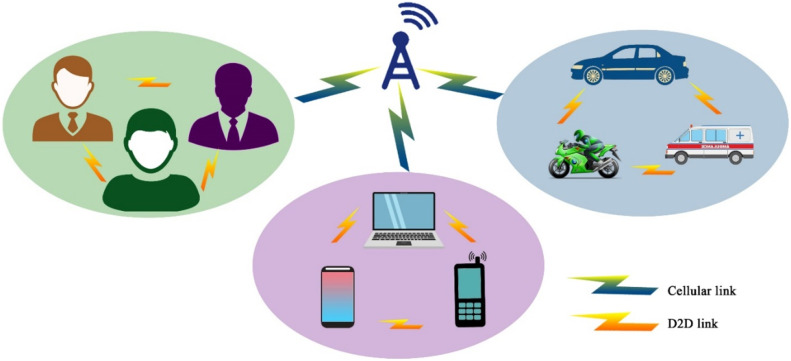
D2D transmission in IoT based networks scenario.

A central UAV-based D2D Communication concept is depicted with surrounding labels indicating the various domains, such as 5G, Cellular, IoT, Power Allocation, Secure D2D, Interference Mitigation, and Military. This visualization illustrates the interconnectedness of UAVs and their role in enhancing communication in these heterogeneous networks. The UAV, positioned above the core communication concept, serves as the focal point in the system, linking various domains through direct device-to-device transmission.

Understanding the specific steps involved in UAV-to-UAV communication is key to appreciating the efficiency of D2D communication. The process begins when one UAV initiates communication and continues through several stages, including bandwidth evaluation, interference management, and power optimization. The flowchart below illustrates this sequence, providing a clear and concise view of how UAVs manage their communication, especially in power-constrained and interference-prone environments [9].

## Network architecture for UAVs in 5G

The integration of UAVs in 5G networks for D2D communication plays a pivotal role in expanding coverage and enhancing throughput. UAVs serve as mobile base stations or relays, allowing for direct communication between devices and the UAVs, bypassing traditional infrastructure. This network architecture diagram provides a visual representation of how UAVs interact with the 5G network, supporting D2D communication to improve connectivity and network resilience.

This diagram serves as a foundational visualization for understanding how UAVs enhance communication in 5G networks through D2D links, enabling ultra-reliable low-latency communication (URLLC).

## Comparison table of D2D protocols across network domains

Different D2D protocols are employed in UAV-based communication across various network domains, each with its advantages and challenges. The table below provides a comparison of key protocols, helping readers understand the differences in their application to UAV-based D2D transmission. Table 1, Table 2, Table 3, Table 4, Table 5, Table 6, Table 7, Table 8, Table 9, Table 10, Table 11, Table 12, Table 13, Table 14, Table 15

**Table 1:** Comparison of D2D protocols across network domains.

Network Domain	Protocol	Advantages	Challenges
**5G**	Power Allocation	High throughput, low latency	Energy consumption, interference control
**IoT**	Lightweight Cryptography	Secure communication, low power usage	Limited resources, scalability
**Military**	Covert Communication	Secure, reliable in critical environments	Jamming resistance, mode selection
**Ad-Hoc**	Clustering & Multi-hop D2D	Extended coverage, high resilience in dynamic environments	Topology changes, routing complexity

**Table 2:** Literature review of D2D transmission over cellular network.

**References**	**Objective**	**Challenge**	**Proposed Solution**	**Gap**
[11]	Reliable swarm communication in D2D over cellular networks	Control UAV swarm with reliability	Introduce Cellular and D2D transmission between swarm	Spectrum efficiency, limited overheads, and D2D relaying
[15]	D2D transmission over cellular networks for high-performance and allocate resources	Spectrum efficiency and power consumption	Use an uplink cellular network where users themselves act as a relay in D2D links	Interference mitigation, reliability, high altitude, and low latency
[20]	Over cellular network mitigate interference in D2D transmission	By optimizing a target of a system and minimizing the interference	An algorithm to match weighted bipartite to minimize interference and maintain the sum rate of the target at the same time	Reliability, latency, spectrum efficiency, and D2D relaying
[29,47]	D2D transmission over cellular network by using distributed resource allocation	Optimize Throughput	Distribute the sharing of spectrum jointly	Reliability, latency, resource allocation, and energy efficiency
[10]	Full-duplex relaying for power allocation under cellular network	Optimize achievable rate for D2D users	Optimized the transmitted power allocation in a closed-form	Interference mitigation, reliability, and resource allocation
[40]	D2D transmission over cellular network using future things	optimization, security, mobility, scalability, and energy efficiency are all issues with D2D communication	D2D communication problems include resource, and security require new algorithms and approaches.	Addresses potential various domain.
[42]	investigation and evaluation of (D2D) potential	network congestion, interference control, spectrum limitations	improved resource distribution to improve (D2D) communication	underutilization of D2D communication in cellular networks to enhance resource efficient

**Table 3:** Literature review of D2D transmission over 5G Network.

**References**	**Objective**	**Challenge**	**Proposed Solution**	**Gap**
[39]	Discuss challenges, issues, and solutions in a 5G environment	Reduce latency, improve data rates, and maximize system capacity	Advancement in D2D links by introducing discovery process, mode selection, power control, interference mitigation in 5G	Spectrum efficiency and energy consumption
[2,45]	In a 5G wireless network, routing algorithms proposed	Extend coverage of the base station	Network assisted routing and balance the load on demand	Spectrum efficiency and interference mitigation
[13]	Selection of relay in 5G network	Selection of optimal relays to avoid time delay	Cooperative transmission and relay selection techniques which were outbound	Energy consumption and coverage capacity
[28]	Protocols for secure 5G transmission	Overheads in transmission	Standard-based routing protocols to remove overheads	Routing algorithms, security, and data rates
[19]	Interference management in 5G environment	In 5G it is a challenging factor to minimize interference	The survey provides the improvement spectrum efficiency, throughput and reduces interference	Relay section and system capacity
[41]	To create an effective ML-3DIM beamforming system, lower interference, and enhance D2D communication security.	Controlling interference, maximizing resources, maintaining security, and enhancing D2D communication's scalability.	Improving data security through encryption in D2D connection	The limitations or problems with present technologies that the suggested solution seeks to resolve

**Table 4:** Literature review of D2D transmission in public safety scenarios.

**References**	**Objective**	**Challenge**	**Proposed Solution**	**Gap**
[33]	In public safety operating UAVs in a MIMO relaying scheme	A high degree of mobility, real-time adaptive learning	MIMO relaying to guide path and examine uncertainty	Spectral efficiency, energy efficiency, and interference mitigation
[30]	Clustering scheme in public safety	Coverage extending challenge	Clustering scheme in D2D links to provide synchronization and improve coverage	Reduction in interference, 5G, and flexibility
[1,44]	For resilient smart cities, the network recovered using small cells in drones	Design parameters like altitude and number of a base station	An intelligently selected number of a base station and altitude by optimization without impact on any other performance factor	Energy efficiency, spectral efficiency, mobility, and 5G
[16]	In public safety to extend coverage multi-hop and D2D transmission used	Interference from neighboring D2D links	Power control techniques by using standard 3GPP services, and multi hoping to extend coverage	LTE, mobility, and flexibility
[8]	For national security and public safety in D2D transmission	Challenges in LTE networks	Clustering scheme	Coverage extension, mobility, and energy efficiency
[17]	Protocols for connectivity and security in D2D transmission for public safety	LTE cellular networks	By selection of user equipment, a direct link protocol was proposed	Interference, 5G, and flexibility

**Table 5:** Literature review of D2D transmission over military network.

**References**	**Objective**	**Challenge**	**Proposed Solution**	**Gap**
[14]	For sensitive and critical applications in the military, a secure protocol for D2D transmission introduced	Jamming, leakage, and spoofing	Security protocols with perfect forward secrecy	The high-speed data rate, and IoT
[3]	D2D transmission for covert communication by introducing jamming and selection of mode	Hide information from an enemy and protect from attack in sensitive domain	Covert communication to confuse the enemy by introducing false information in the network	Reconnaissance, Privacy/Confidentiality, and IoT
[26]	Block chain-based protocols for the internet of military vehicles environment	Security, privacy, and latency	Block chain-based protocols in IoMV with the use of IPFS	Reconnaissance, and Reduce Jamming/Interference
[4]	Military communication in 5G environment	5G network is an advance and many factors do not support 5G and introduce impacts on privacy and security	By introducing services from standard 3GPP and SA3 groups in a 5G environment	High-Speed Data Rate, Block chain Technology, and IoT
[27]	Block chain-based secure transmission	Disruption in operation and to process data confidentially	D2D transmission based on block chain which provides security in the military domain	Reconnaissance, Covert Capacity, and High-Speed Data Rate
[41]	Secure D2D military communication using AI, anti-jamming, 3D MIMO.	Challenges include key management, computational cost, and side-channel attacks.	Secure implementation, scalability improvement, hardware optimization, and effective key management	Device-to-device communication over military networks is not safe or effective.

**Table 6:** Literature review of D2D transmission over social network.

**References**	**Objective**	**Challenge**	**Proposed Solution**	**Gap**
[32]	Avoid cascading failure by the placement autonomous relay in the social domain	Node resilience	Increase number of links, avoid failure in cascading, an autonomous device placement was proposed, minimize node resilience by optimization and no complete information required for the placement	Traffic offloading and IoT
[22]	A social network in D2D transmission	Throughputs, latency, and spectral efficiency	Use social IoT where people and things are involved together in networks and assisted autonomously	Interference and power optimization
[38]	UAV-based D2D transmission depends upon the social domain	The problem is NP-hard and could not track	By power optimization and maintaining the quality of service (QoS), and minimizing the social group activity by summation and hidden non-convex components were also introduced	Autonomous devices, relaying, and traffic offloading
[35]	In a wireless domain, D2D links introduce social networks	Wireless networks losses	Optimization of D2D by introducing physical and social network layers and the algorithm offload traffic	Physical interference, autonomous, and relaying

**Table 7:** Literature review of D2D transmission over Ad-Hoc network.

**References**	**Objective**	**Challenge**	**Proposed Solution**	**Gap**
[24]	Challenges and applications in the wireless network a flying ad-hoc perspective introduced	If a nation allows flying autonomously in the sky, then the sky was crowded with UAVs	The new technique of FANET was introduced, easy to deploy, robust, and reliable	LTE, VANET, MANET, and DTN
[7]	Ad-hoc network for VANET	Network deployment in an emergency	Emergency VANET, a lightweight, small network to provide fully autonomous, multi-tasking, and ultra-compact LTE cells enable the mobile network to smoothly	FANET, 5G, and security
[23]	Cluster-based VANET in safety scenarios	Message exchange in VANET safely	Cluster-based D2D transmission give high-data rates delivery	LTE and clustering
[37]	Ad-hoc based D2D transmission in cellular network	Communication with each other in mobile networks over a licensed frequency	Ad-hoc based network deployed included DTN	LTE, VANET, and privacy

**Table 8:** Literature review of D2D transmission in IoT based network.

**References**	**Objective**	**Challenge**	**Proposed Solution**	**Gap**
[31]	5G IoT network based on cryptography to secure D2D transmission	Security and privacy challenges in IoT devices due to less available resources	Lightweight cryptography gives very secure authentication and data remained confidential	NOMA, emergency communication, public safety networks, and mobility
[21]	In heterogeneous IoT, an energy-efficient transmission based on UAV was performed sing DSF-NOMA scheme	Power consumption challenges	Non-orthogonal multiple access (NOMA) scheme proposed to reduce power consumption and jointly tuned by the user using the scheme	5G, mobility, and energy efficiency
[34]	UAV-based adaptive learning to make the system energy efficient in the wireless IoT domain	The role of nodes be critical in terms of position in 3D	Optimize problem of maximizing and a distributed power in IoT node was adopted by a non-cooperative game through an adaptive approach	Extended coverage, 5G, and OFDMA
[25]	UAV IoT coverage in natural or human-made disaster, a transceiver was designed and a concept of multi-hop D2D concept was presented	In a disastrous location, the coverage based on IoT damaged severely	Multi-hop D2D provides reliable transmission and an array of antenna-based transceivers designed to optimize the performance of the UAV	NOMA, OFDMA, and energy efficiency

**Table 9:** Comparison of key features of D2D transmission over cellular networks.

**UAV-Based D2D Communication over Cellular Networking Features**	[11]	[29,47]	[10]	[15]	[20]
Interference Mitigation	✓	✓	✗	✗	✓
High Reliability	✓	✗	✗	✗	✗
Low Latency	✓	✗	✗	✗	✗
High Altitude	✓	✗	✗	✗	✗
Spectrum Efficiency	✗	✓	✓	✓	✗
Power Optimization	✓	✓	✓	✓	✗
Energy Efficiency	✗	✗	✓	✗	✗
Limited Overheads	✗	✓	✗	✗	✗
D2D Relaying	✗	✗	✓	✓	✗
Achievable Rate	✗	✗	Maximum	Average	✗
LTE,4G,5G	✗	✗	✗	✗	✓
Resource Allocation	✗	✗	✗	✗	✓

**Table 10:** Comparison of key features of D2D transmission over 5G networks.

**UAV-Based D2D Communication under 5G Networks Features**	[19,46]	[28]	[2,45]	[13]	[39]
Spectrum Efficiency	✓	✗	✗	✓	✗
Low Energy Consumption	✓	✗	✗	✗	✗
Maximum Throughput	✓	✗	✗	✗	✗
Coverage Enhancement	✓	✓	✓	✓	✗
Reduced end-to-end Latency	✓	✗	✗	✗	✗
Interference Management	✓	✗	✗	✗	✓
Low Computational Time	✗	✓	✗	✗	✗
Key Exchange Protocol	✗	✓	✗	✗	✗
Routing Algorithms	✗	✗	✓	✗	✗
Relay Selection	✗	✗	✗	✓	✗
Improved Data Rates	✗	✗	✗	✗	✓
System Capacity	✗	✗	✗	✗	✓
Security	✗	✗	✗	✗	✓

**Table 11:** Comparison of key features of D2D transmission in Public safety scenarios.

**Public Safety UAV-Based D2D Communication Features**	[16]	[8]	[17]	[1,44]	[30]	[33]
Coverage Extension	✓	✗	✓	✓	✓	✓
Public Safety	✓	✓	✓	✓	✓	✓
HetNets	✓	✗	✗	✗	✗	✗
Energy Efficiency	✓	✗	✗	✗	✓	✗
Spectral Efficiency	✓	✗	✗	✗	✗	✗
Reduce Interference	✓	✗	✗	✗	✗	✗
LTE	✗	✓	✓	✗	✗	✓
Clustering	✗	✓	✗	✗	✓	✗
5G	✗	✓	✗	✗	✗	✓
Mobility	✗	✗	✗	✗	✓	✓
SINR	✗	✗	✗	✗	✓	✗
Flexibility	✗	✗	✗	✗	✗	✓
MIMO System	✗	✗	✗	✗	✗	✓
3GPP	✓	✗	✗	✗	✗	✓

**Table 12:** Comparison of key features of D2D transmission over military networks.

**UAV-Based D2D Networking for Military Communication Features**	[14]	[27]	[4]	[3]	[26]
Reconnaissance	✓	✗	✗	✗	✗
Secure Communication	✓	✓	✓	✓	✓
Privacy/Confidentiality	✓	✗	✗	✗	✓
Reduce Jamming/Interference	✓	✗	✗	✓	✗
Covert Capacity	✗	✗	✗	✓	✗
High-Speed Data Rate	✗	✗	✗	✓	✓
Blockchain Technology	✗	✓	✗	✗	✓
IoT	✗	✓	✗	✗	✓
3GPP(5G)	✗	✗	✓	✗	✗
Mobile Edge Computing	✗	✓	✗	✗	✗

**Table 13:** Comparison of key features of D2D transmission over social network.

**UAV-Based D2D Communication for Social Networking Features**	[38]	[22]	[32]	[35]
Power Optimization	✓	✗	✓	✓
Physical Interference	✓	✗	✗	✗
Convex Optimization	✓	✗	✗	✗
Social IoT	✗	✓	✗	✗
Autonomous Devices	✗	✓	✓	✗
Relaying	✗	✗	✓	✗
Traffic Offloading	✗	✓	✗	✓

**Table 14:** Comparison of key features of D2D transmission over Ad-Hoc networks.

**UAV-Based D2D Communication in Ad-Hoc Networks Features**	[7]	[24]	[37]	[23]
LTE	✓	✗	✗	✓
VANET	✓	✗	✗	✓
MANET	✓	✗	✓	✗
FANET	✗	✓	✗	✗
5G,6G	✗	✓	✗	✗
Security/Privacy	✗	✓	✗	✓
Delay Tolerant Network	✗	✗	✓	✗
Clustering	✗	✗	✗	✓
Software Radio	✓	✗	✗	✗

**Table 15:** Comparison of key features of D2D transmission in IoT based network.

**UAV-Based D2D Communication in IoT** **Features**	[21]	[34]	[25]	[31,47]
Het-IoT	✓	✗	✗	✗
NOMA	✓	✓	✗	✗
OFDMA	✓	✗	✗	✗
Emergency Communication	✓	✗	✓	✗
IoT	✓	✓	✓	✓
Energy Efficiency	✗	✓	✗	✗
Public Safety Networks	✗	✓	✓	✗
Extend Coverage	✗	✗	✓	✓
Mobility	✗	✓	✓	✗
D2D Links	✓	✓	✓	✓
5G Architecture	✗	✗	✓	✗
Lightweight Cryptography	✗	✗	✗	✓

This table highlights how protocols such as power allocation in 5G, lightweight cryptography in IoT, and clustering in ad-hoc networks are applied to D2D communication, focusing on their respective advantages and challenges.

## Key contributions of the study

This paper makes several significant contributions to the field of UAV-based D2D Communication within heterogeneous networks, specifically focusing on 5G, IoT, military, cellular, and ad-hoc networks. The key contributions are as follows:

## Comprehensive review of UAV-based D2D communication across multiple domains

The paper provides an in-depth review of UAV-based D2D communication across multiple network domains such as 5G, military, IoT, ad-hoc, and cellular networks, addressing their unique requirements and challenges in the context of UAV communications.

It explores the implications of UAVs enhancing communication reliability, latency reduction, and network flexibility in these heterogeneous environments.

## Analysis of power allocation and interference mitigation techniques

The paper delves into the power allocation strategies and interference mitigation techniques critical for the effective functioning of D2D communication in UAV networks.

It identifies key challenges in power optimization and interference management and suggests methods for improving efficiency and minimizing interference in real-world UAV-based D2D communication systems.

## Security framework for UAV-based D2D communication

The research proposes a security framework tailored for UAV-based D2D networks, focusing on ensuring secure communication across different domains, particularly in the military and critical infrastructure sectors.

The study addresses challenges such as jamming, data interception, and spoofing, and presents solutions like lightweight cryptography and blockchain-based authentication to safeguard data integrity and privacy.

## Integration of UAVs in 5G networks for enhanced D2D communication

The study evaluates the integration of UAVs within 5G networks to enhance D2D communication. This includes UAVs acting as mobile base stations or relays to facilitate direct communication between devices, improving network reliability and coverage.

The paper also discusses how UAVs can support ultra-reliable low-latency communication (URLLC) for applications like smart cities and emergency response systems.

## Proposed methodologies for efficient resource allocation in UAV networks

The paper introduces new methodologies for efficient resource allocation and dynamic spectrum management in UAV-based D2D networks.

It highlights the potential for AI-driven algorithms and machine learning techniques to optimize network resources in real-time, allowing for better management of UAV fleets and network resources.

This paper also described undermentioned research questions, analysis and find the answered. Details of RQs are as follows:

**RQ 1**: How does device-to-device (D2D) communication perform across different types of wireless networks (e.g., 4G, 5G, Wi-Fi, LoRaWAN)?
**RQ 2**: What are the primary security and privacy challenges faced in D2D transmission over heterogeneous networks?
**RQ 3**: What are the solutions of security and privacy in D2D transmission over heterogeneous networks?

At the end of this paper, open research topics are provided which are the most common and important to be resolved before establishing an efficient D2D link in UAVs. The D2D transmission is completely discussed with opportunities and further research topics are also provided in this paper. In short, this paper is a complete package of UAV-based D2D transmission over multiple networks. The UAV-based D2D transmission is divided into seven different heads. They are presented in a flow chart given below;

## Literature review

Device-to-Device (D2D) networks improve spectral efficiency and lower latency by allowing direct data transmission between adjacent devices without requiring base station routing. By offloading traffic, D2D improves throughput in cellular networks. Because it facilitates ultra-reliable low-latency communication (URLLC), it is essential to 5G. In times of crisis or in situations without infrastructure, D2D guarantees strong and reliable connectivity for military and public safety communications. Local content sharing helps social networks, and D2D allows ad hoc networks to establish dynamic peer-to-peer connections. D2D makes smart devices in the Internet of Things (IoT) capable of effective communication, facilitating real-time data sharing and cooperative actions. Details of Device-to-Device (D2D) networks are as follows:

**RQ 1:** How does device-to-device (D2D) communication perform across different types of wireless networks (e.g., 4G, 5G, Wi-Fi, LoRaWAN)?

### D2D over cellular networks

**In 2020**, a concept of swarm using UAV with D2D links was introduced over a cellular network [11]. **In 2018** for D2D transmission over cellular networks was carried out collectively with the help of D2D pairs. Many D2D users and relay devices were used [15]. **In 2017**, by decreasing sum rate, interference in D2D transmission was mitigated [20]. **In 2013**, D2D transmission over the cellular network a distributed allocation for the resource was done to limit the overheads [29]. Full-duplex relaying for power allocation in D2D transmission over cellular networks was proposed in the same year [10]. **In 2023,** a concept was discussed that future cellular networks' capacity and efficiency can only be increased with D2D communication.

Networks [40]. Cellular networks are covered in this paper in relation to D2D communication, in which devices speak with one another directly to lower network load and boost efficiency [42].

### D2D over 5G networks

**In 2020**, the challenges, issues, and solutions were discussed in the 5G environment for D2D transmission between UAVs [39]. **In 2018**, distributed routing algorithms for D2D transmission over cellular networks were proposed in a 5G environment to enhance the coverage [2]. **In 2017**, to increase spectrum efficiency of the cellular network in D2D transmission in a 5G environment was presented to extend the broadcasting [13].**In 2016**, some key protocols were designed for safe D2D transmission in a 5G environment [28]. In 5G network research to mitigate interference was also carried out and solutions for secure transmission were proposed [19].

In 2023 studied that 5G, D2D communication improves massive machine-type communication (mMTC), ultra-reliable low-latency communication (URLLC), and broadband services [40].

In 2022, a concept was introducing that the advanced encryption standards improve data security in D2D communication, while 3D MIMO beamforming combined with the improved support vector machine method improves throughput, signal to interference plus noise ratio (SINR), and signal to noise ratio (SNR) in 5G networks [41]. In 2023, Comparing 5G networks to earlier network generations to facilitate effective Device-to-Device (D2D) communication by providing faster data speeds, reduced latency, and better resource management [42].

### D2D in public safety scenarios

**In 2021**, UAVs were used D2D transmission in public safety scenarios, for this purpose a technique of MIMO relaying was proposed [33]. **In 2018**, a clustering scheme was proposed to expand coverage for public safety [30]. **In 2016**, for the recovery of the networks in resilient smart cities after a disastrous attack, a drone with a small cellular network was empowered to provide connectivity [1]. **In2015**, to increase the coverage in the disastrous areas, UAV sues multi hoping to mitigate interference and minimize power consumption [16].

In 2022, during network overloads or breakdowns, D2D communication guarantees dependable, direct coordination among emergency personnel [40].

### D2D over military networks

**In 2021**, in the period of war jamming and selection of mode was very important, to hide sensitive information from the enemy, many protocols were proposed to secure the information during transmission in the military domain [14]. To hide information and to confuse the enemy false information was introduced in the network and a concept of covert communication was proposed [3]. In the same year, block chain technology was proposed which was designed beyond 5G for military purposes, and a block chain-based onion protocol for the internet of military vehicles [26,27]. **In 2020**, 5G was first introduced in the military domain and focused on the security of information using services by 3GPP and SA3 groups [4]. In 2022, D2D communication must be secure and flexible for military networks. 3D MIMO increases the precision of the signal. ML improves connections and identifies jamming. Strengthening Learning improves the usage of spectrum [41].

### D2D over social networks

**In 2021**, when there was a cascading failure using social networks, autonomous relaying routing algorithms and other optimization techniques were proposed [32]. **In 2019**, UAVs using D2D links were also used in IoT in a 5G environment, and to make a network secured, a concept of lightweight cryptography was proposed [22].**In 2018**, by optimizing power and by convex optimization in the social domain, D2D transmission between UAVs was performed [38]. **In 2015**, cumulative distribution function (CDF) was applied in the social domain for D2D transmission between UAVs for optimization in global wireless networks [35].

### D2D over ad-hoc networks

**In 2020**, the concept of FANET was presented to enable wireless technologies, applications, and challenges [24]. **In 2018**, the ad-hoc cellular network deployed for VANET which was lightweight LTE networks, software radio, and open-source software were used [7]. In the same year, safety was taken in VANET using D2D transmission in a cluster form [23]. **In 2016**, the ad-hoc based D2D transmission scheme of Delay Tolerant Network (DTN) was proposed [37].

### D2D in IoT based networks

**In 2019,** as the IoT-based devices were constrained in resource allocation so, the security issues become more crucial, the scheme was proposed to offload traffic [31]. **In 2018,** in Het-IoT UAV guided in emergency transmission using DSF-NOMA scheme [21]. In the disastrous areas, the coverage was extended by designing transceivers for D2D transmission in UAVs [34]. In the same year, using adaptive learning a wireless network for public safety in the IoT-based domain was proposed [25]. In 2023, D2D communication lessens dependency on centralized cloud infrastructure by enabling low-latency, direct connectivity and data transmission across adjacent IoT devices [40].

## UAV-based D2D communication over cellular networks

UAV-based D2D communication over a cellular network is the network in which the gadgets communicate with each other using D2D links and the main network to each device is from the base station which is provided by a cellular link. The concept is further explained in the figure below:

This diagram illustrates the fundamental concept of Device-to-Device (D2D) communication within a cellular network. It highlights the direct communication between devices in proximity, bypassing the central network infrastructure. This visual represents the key components involved, such as the user equipment (UE), base stations, and cellular towers, focusing on the efficiency and latency improvements offered by D2D communication.

This scenario-based diagram shows a practical implementation of D2D communication in a cellular network. It depicts how devices within a cellular network can establish direct communication links with one another, optimizing resources and reducing network congestion. The diagram demonstrates various aspects like interference management and handover between D2D and cellular communication.

### Swarm communication

Cellular Networks have existence with a problem that is difficult to interface and command a swarm of drones with accuracy and privacy. As the Swarm of a drone is functioning at a higher elevation angle and robust G2A channels, it is substantially revealed to numerous (GBS), during the GBSs that are helping end-users on the ground (inhabited GBSs) initiate powerful obstruction to the swarm of a drone. To minimize the noise, a two-state communication protocol by using cellular and (D2D) transmission for the swarm of drones is proposed. During State I, one swarm head is selected for G2A channel evaluation, and all other GBSs that are not providing services to users on the ground (handy GBSs) broadcast an ordinary standard message to the swarm of drones concurrently, by operating the similar cellular frequency band. The head of a swarm along with other members of the swarm can employ the high-potential gain from many accessible GBSs’ communication, to counter the powerful interference from inhabited GBSs, whereas few drones can fail to decrypt the message because of unconnected G2A channels. In-State II, all the drones which decrypted the ordinary standard message in State I relay more extremely to the other drones in the swarm over D2D communication, by introducing the minimum obstruction in a D2D frequency band and the vicinity surrounded by drones [11].

### Resource allocation

The distributed connected spectrum participating and power issuance technique is offered to enhance the particular resources in the (D2D) transmission under cellular networks (CNs) with restricted beckoning in flight. The technique focuses to improve the throughput of D2D transmissions over the combined uplink frequency spectrum, for the time being, the cellular users (CUs) are protected by a base station (BS) from the noise of D2D users. Stackelberg game (SG) is produced to design the issue for obtaining the distributed technique. In the game, the BS, as a commander, correlates the noise from the D2D users to the cellular uplinks and boosts its benefits by estimating the noise. Moreover, D2D couples, as companions, be a competitor for the spectrum as a Nash incompliant game to boost their data rates independently. The designing implementation at the BS connecting to the Stackelberg Equilibrium is considered. In the end, numeric models are introduced to demonstrate the preferred technique. The technique is essential on the resource issuing and the Cellular User security with restricted beckoning in flight [29,47].

#### Performance analysis

Combined D2D transmission in an uplink cellular network where D2D users act as relays for cellular users. We obtain the interruption of service possibility of a cellular end-user and the intermediate attainable rate via D2D sender in respect to a D2D recipient in detailed configuration. We achieve feasible range and energy issuing to extend the complete mean attainable rate receiving the shutdown possibility restrictions [15].

### Power allocation

D2D links permit direct communications connecting two closes (UEs), which boost ordinary facilities in cellular networks. Observing full-duplex (FD) relaying can attain excessive spectrum and effective power compared to half-duplex (HD) relaying, an advanced D2D transmission technique permits D2D connection to basic cellular downlink allocating D2D senders as FD relays to serve cellular downlink communications is proposed. The network goal is to enhance the feasible rates for the users in D2D while attaining the lowest rate demands of the cellular users. Consequently, first, acquire the rates attainable for the D2D users as well as for the cellular users. Subsequently, we execute the goal by enhancing the energy transmitting in the vicinity of a base station (BS) and the D2D sender. Under the composite energy restriction, we can resolve the effective energy assignment issue in the bounded region [10].

### Interference minimization in D2D communication underlying cellular networks

Interference minimization while sustaining a networking goal aggregate rate by a distribution of radio receivers between cellular end-user hardware and (D2D) couples is the foremost investigation in long term evolution (LTE) with far away (4G and 5G). Complete network aggregate rate referring to a cellular system could be made better whether cellular end-user hardware and D2D couples distributed source factors. Nevertheless, various distributions as well reduce based on aggregate rate and rise network noise. Given the indicated examination, we discourse two kinds about functions (clear and confined) in source issuing in favor of noise reduction source issuing complication. We present a two-stage source issuing method towards the two clear and confined functions, where the purpose intends to reduce the network noise and simultaneously, sustaining a goal of a network aggregate rate. In phase-I of the suggested method, a weighted dual coordinated method was applied to mitigate the noise and achieve a reasonable elementary result. In certain instances, noise can be reduced which is initiated in stage-I of the method. Consequently, in stage-II, ordinary exploring methods are used to upgrade the result. These methods are two-phase auction-based fair and interference aware resource allocation algorithm (ZAFIRA). We differentiate this method with a minimum knapsack-based interference resource allocation algorithm (MIKIRA) [20].

### Comparison of D2D communication underlying cellular network

Under cellular networks, device-to-device (D2D) communication improves spectrum efficiency and lowers latency by enabling direct communication between adjacent devices without going via the base station. D2D allows proximity-based services and provides greater resource utilization than regular cellular communication. It does, however, encounter difficulties with resource allocation and interference control. Its performance is improved by integration with 4G and 5G networks, although sophisticated coordination methods are needed. All things considered, D2D in cellular settings offers a viable way to communicate effectively, low-latency, and at high throughput in situations involving dense networks. Details are given below:

## D2D communication under 5G networks

UAV based D2D transmission is performed between the devices under 5G networks in which wireless beam is provided to gadgets within a 5G boundary which is shown in the figure below;

This diagram depicts the D2D communication in the context of 5G networks, where UAVs act as intermediaries to improve latency and data throughput. The visual emphasizes the benefits of ultra-reliable low-latency communication (URLLC) and massive machine-type communication (mMTC) in 5G, showing how D2D links enhance the overall network capacity.

This figure presents the application of D2D communication in public safety scenarios, showing how UAVs enable ad hoc networks for rapid communication in disaster areas. The diagram demonstrates how UAVs facilitate direct communication between responders and devices in the absence of infrastructure, improving operational efficiency in emergency communication.

### Interference management

D2D transmission is an auspicious idea to intensify the production of gadgets by permitting straight through communication allying nearly discovered couples of users. The primary work has shown that straight-through transmissions are bound to upgrade bandwidth reutilize, performance, power loss, broadcast, and minimize persistent jitter. Furthermore, this tends to empower the modeling of the latest P2P servicing and position-based applications. Consequently, recent search tendency has disclosed that D2D intended particular technologies in upcoming inception cellular system, which is 5G. Nevertheless, establishing a D2D cellular system obtrudes numerous high-tech provocations. Noise conduction in cellular users and D2D users is contemplated to ensure the most condemnatory challenge when D2D is established to the cellular system due to D2D users using a similarly licensed bandwidth with cellular users. A complete and detailed study about several modern perspectives related to noise occurrence in D2D transmission empowered in cellular systems. To manage interference in D2D transmission over 5G, then 5G cellular systems are presented [19].

The future 5G communication network required high bandwidth to achieve greater data, and it is possible by the deployment of small cells in the distance of 200 meters of radius. As a result, high data rates along with low signal delay will be achieved. However, numerous issues arise with the strategy as follows;

• Intercell interference
• Intracell interference

#### Intercell interference

Intercell interference is caused when different users try to use the same resource at the same time. In drone communication, inter-cell interference integration approaches reflect limitations to the radio resource management (RRM)block, (which is the model driven management of the interference of the channel on same frequency and further transmission features in wireless systems), boost supportive channel state over member of users that are critically affected by the interference, and consequently achieving high spectral efficiency [46].

In the figure below, interference between user equipment I (UE 1), user equipment II (UE 2), and user equipment III (UE 3) is shown where strong interference is located between UE 1 and UE 2 and weak interference is clearly visible between UE 1 and UE 2 from base-station 3 (Site 3) while base-station 2 (Site 2) and base-station 3 (Site 3) creates weak interference for UE 3. RRM generates inter-cell signaling to raise channel conditions and attain high frequency.

#### Intracellular interference

Device to device links causes interference between cellular and D2D users, which reaches to the result to create intra-cell interference. The intra cell interference is shown in the figure given,

### Secure protocols for communication

(D2D) transmission connecting two end-user tools (UTs) within a cellular network. Towards the indicated framework, on the condition that the pair of the end-user tools be within system broadcast, internet protocols in favor of D2D transmission are presented. The protocols derived from the standard Diffie-Hellman (DH) derive from a public key and further secret writing activities. A comprehensive risk investigation towards the protocols also revealed flexibility opposite to identifying the theft. Moreover, along with analytical outcomes, manifest with the intent that protocols possess low computational time and low aerial transmission corresponding in the company of present internet protocols for D2D transmission [28]. The protocols exist nearly executable and most acceptable conductive to D2D transmission over 5G cellular networks.

### Routing algorithms

This paper is regarded as a System Assisted Routing method to get D2D transmission within 5G cellular constructions accompanied by an objective to increase the broadcast of BSs. System Assisted Routing to consider that D2D transmissions in cellular networks cope at BS. The Burden Sharing Build Precise Ad Hoc as required Multichannel range. Vector method reviewed in the guise of recommendation [2]. The two methods exhibit enhancement of 35% in power-efficient with an extension of 15% in the number of data delivered.

### Relay selection

D2D transmissions carried in the direction of the cellular network have newly been presented to enhance bandwidth effectiveness and extension of broadcast. The relay option takes part in the primary part of collaborative systems. In D2D transmission, whether the selected relay is neither the greatest relay, at that time the entire transmission does not tend to be victorious along with the source point in the direction of the goal point. In addition to selecting the adequate relays, on condition that additional response and waiting period have existence between source and relay points subsequently it guides to loss of bandwidth effectiveness. A study to choose the relay methods desirable considering D2D transmission over a 5G cellular system is proposed [13].

The first case described the communication between gadgets fully based on base station in the areas where broadcasting is very poor. One gadget work as a relay which is close to BS and full control is in the hand of BS.

The second case is the straightforward communication between the transmitter and the receiver command from BS, the method as noise occurrences.

Case three shows the communication between the transmitter and receiver commanded by the relay. Here noise occurrence depends on the gadget which acts as a relay.

The last case shows straightforward communication among the gadgets having no control from BS.

**RQ 2:** What are the primary security and privacy challenges faced in D2D transmission over heterogeneous networks?

### 5G Environment: issues, solutions, and challenges

(D2D) transmission turns out the latest extent via versatile territory, relaxing about data interchange operation linking substantially adjacent gadgets. In respect to attain a productive exertion referring to accessible sources, decrease bandwidth, upgrade data rates, and improve network scope, D2D transmission put to use neighboring transmitting gadgets. Appropriate to mobile driver’s operation to accumulate the tactical transmissions conducive to preservation as far as proximity-based assistances and refine the production of the system operates the progress of D2D. This paper proposed an immense analysis of presented solutions designed to intensify the privacy in D2D transmission. The primary objective of the analysis is to contemporize a vast evaluation of the latest up gradation in several D2D territories similarly the recognition procedure, model selection techniques, noise control, energy command methods, and ultimately the mode choosing for D2D implementations towards 5G technologies. Furthermore, we focus attention on the wide-open issues and recognize the provocation admire to the D2D transmission issue [39].

#### Video streaming

**Decrease Latency:** While human reaction time is more than 200 milliseconds, 5G is expected to send and receive information in one millisecond or even less. When it comes to streaming, this means that digital objects will be able to replicate real-time interactions. This is proof in how 5G provides massive enhancement to live streaming. 5G is providing 10x decrease in end-to-end latency. The increased speed improves the production of live-streaming on devices.

#### M2M (Mobile To Mobile)

M2M networks, with their massive deployments, will provide new opportunities to 5G operators. The speed, reliability, flexibility, and scalability provided by 5G services will be a enabler of M2M technology and spread over all domains.

#### Emergency networks

5G will present a great advancement for people working in the emergency services, as it will enable technological deployment (drones) in public safety.

#### Voice and data

In 5G, the default voice codec in 5G enables “HD voice+” using the 3GPP standardized Enhanced Voice Services (EVS).

### Comparison of D2D communication underlying 5G network

Comparing 5G networks to earlier generations, device-to-device (D2D) connectivity delivers quicker data interchange, reduced latency, and improved dependability. Without going via base stations, it allows direct communication between adjacent devices, improving network efficiency, facilitating widespread IoT connection, and opening up vital applications in smart cities, public safety, and vehicle networks. Details are given below:

**RQ 3:** What are the solutions of security and privacy in D2D transmission over heterogeneous networks?

## UAV-based D2D solutions for public safety

UAV-based D2D transmission in public safety plays a vital role. In the figure below there is a fire burn in the pizza hut then to provide an immediate rescue UAVs provide information to the base station for an emergency by obtaining information from VANET and provide to the swarm of drones which are then transmitted to a relay drone to provide information of disaster to the base station.

This diagram focuses on the public safety applications of UAV-based D2D communication. It illustrates how UAVs are deployed in emergency situations, such as search and rescue operations or disaster recovery, to provide secure, low-latency communication links between first responders, ground stations, and other devices.

This figure presents the application of D2D communication in public safety scenarios, showing how UAVs enable ad hoc networks for rapid communication in disaster areas. The diagram demonstrates how UAVs facilitate direct communication between responders and devices in the absence of infrastructure, improving operational efficiency in emergency communication.

### Extending coverage

We review D2D transmissions operate to broaden the broadcast location regarding agile main central stations for public safety scenarios escorted by limited broadcast. A trainer is designed based on a 3GPP licensed cooperative network standard-compliant system for D2D and diverse system technicalities gave general security. The trainer estimates the production of D2D multiple transmissions elementary cellular systems as the occasion of limited broadcast. Enhancements in power and bandwidth effectiveness, when differentiated as well as the scenario of straightforward end-user tools to central station transmissions, are indicated. Furthermore, the end-user tool's energy command topologies are used to minimize the consequences of noise from adjacent D2D connections [16].

### National security

D2D transmissions have been suggested as fundamental to Long Term Evolution (LTE) networks as a source of gathering the accessibility, reutilizing, and hop gains. Nevertheless, D2D transmissions as well are in the service as a technical element for giving general security and catastrophe consolation, governmental safety, and general security services. In the United States, for instance, bandwidth is reserved in the 700 MHz frequency considering an LTE-based general security system. The essential condition for developing a wide range of general defense and accidental reassurance (GDAR) and governmental safety and general security (GSGS) serves efficient networks to give an approach to cellular networks at the time when a base station is obtainable and to effectively contribute to public help though a subcategory or every of the system points embellishes malfunction because of general catastrophe or contingency. We evaluate various essential demands, technical issues, and key addresses that have to be adjusted in place to empower LTE systems and specific D2D transmissions to meet up with general defense and accidental reassurance (GDAR) and governmental safety and general security (GSGS)-concerned demands. Generally, a congregating-method-dependent detain to the layout of a device network that consolidates cellular and ad-hoc working method rely on the accessibility of base station points is presented. The presented technique is nowadays reviewed as a technical tool of the progressing 5G idea evolved by the European 5G research project METIS [8].

### Connectivity and security

D2D transmission is the main characteristic for numerous types of mobile systems and specifically for the Advanced LTE cellular network, wherever a fundamental system is designed among end-user tools independently a developed point B. The probability to permit straightforward connections amongst end-user tools participates in a significant role when the commercialized LTE base station is liable to defeat or enhance inaccessibility behind a tragedy. For general security, a balloon is flying high has a 4G network with is to re-establish the short-term connection. For all circumstances where end-user tools are out of the range of node B like inside, D2D becomes critical. We presented to create straightforward connections make use of transmission protocol which depend on the communication framework sent in a transmitting manner by elected end-user tools. We search into the privacy features of this D2D treaty appropriate for general security end-users with out-of-range end-user tools dependent on participating encoder. We trace a system affinity evaluation of the protected agreement through which we can be seen the existing compensation among connectivity and the rising in flight attached with safety for an unlike gain of the network specifications [17].

### Disaster recovery

Strong transmission systems, which can proceed with functions despite a disaster, have the main characteristics of upcoming digitally connected cities. The current breeding of UAVs thrust by the accessibility of low-cost product hardware gives an advanced approach for equipping such systems. Specifically, the arrival of such drone-based social media and google projects small-scale cellular systems are not a dream. Small-scale cellular systems are irresistible for general security systems due to their immediate implementation ability and inherent system evaluability. While small-scale cellular systems have achieved little awareness recently, the configuration of systems not used comprehensive pass over. Specifically, the order of such systems with the functional ground cellular system in a post misadventure scenario was not inquired [1]. Furthermore, layout framework as a feasible height and quantity of UAV central stations, for damaged central stations, transmitting environment, have not been traversed. To overcome the discussed challenges, we propose detailed parameters which were designed from a probabilistic stance. We then recruit the evolved parameter to inquire about the effect of various parametrized adaptations on the presentation of the small-scale cellular systems. Beyond dropping any indefinite statement, the achievement is the broadcast possibility of a narrowband flying user. It is illustrated that by logically choosing the quantity of UAVs and their correlated heights, broadcasting increased with the use of a ground base station [44].

### Clustering

General security transmission gives efficient transmission between the foremost replier and sufferer in the general protection framework. D2D transmission is a scheme that increases broadcasting in cellular systems. We presented a novel D2D assembling scheme to enlarge cellular broadcasting for general security transmission. In the presented technique, assemble forefronts are chosen from a gathering of general security end-user tools depending upon various benchmarks like persisting energy of a cell, signal to interference plus noise ratio, a quantity of immobile gadgets locations, and mobility. All assemble forefront gives synchronism, transmission resource statics conduction, and broadcast to its cluster partners [30].

### MIMO relaying UAVs

Techniques to execute transmission in the unsophisticated and man-made catastrophe were extensively debated in the technological group. Researchers trust that the drone relays do take part in a condemnatory role in 5G general security transmission (GST) because of scientific supremacy. They contain numerous remarkable benefits like intensive maneuverability, resilience, extraordinary LOS, concurrent adaptable path organization. This paper proposed connection development in the 3G partnership program standard related to the employment of D2D schemes which utilize LTE transmission systems, possible augmentation for 5G, and research on the effect of cellular flexibility on relay drones operating by using software network simulator 3. The paper also presented MIMO transmission put in an application of UAV and gives the latest path to guide end users go through undesirable situations. All transmission procedures are based on UAV speed and the way to employ MIMO relays [33].

### Comparison of D2D communication in public safety scenarios

Device-to-Device (D2D) communication, which allows devices to exchange data directly without depending on centralized infrastructure, is essential in public safety scenarios. When it comes to crises or network disruptions, D2D provides better resilience, faster reaction times, and more dependability than standard cellular systems. For disaster recovery, search and rescue, and first responder coordination in dynamic and infrastructure-constrained contexts, this makes it perfect. Details are tabulated below:

## UAV-based D2D solutions for military communication

UAV-based Device-to-Device (D2D) transmission is essential for improving military networks' connectivity, particularly in dangerous or isolated areas. These solutions provide reliable and low-latency data sharing by allowing direct contact between ground units and UAVs without depending on centralized infrastructure. UAVs can enhance real-time situational awareness and mission coordination by serving as mobile relays or data collectors. By using this method, network coverage is increased, communication delays are decreased, and operational efficiency is raised. In dynamic and uncertain combat situations, UAVs' versatility and mobility provide them a strategic advantage. Details of UAV based D2D transmission over military networks is given in the figure below;

This diagram illustrates the use of UAVs in military communication via D2D transmission, providing a tactical advantage by enabling real-time, secure communication in areas with minimal infrastructure. UAVs enhance operational reliability and security, ensuring secure communication for defense applications.

This visual presents D2D communication in a military network, showcasing how UAVs and devices communicate directly for secure, low-latency exchange of critical information during operations. The diagram highlights aspects like signal encryption, interference avoidance, and range extension crucial in military settings.

### Secure military communication

Drones take part in the most important role in several paddocks like military, aerospace, exploration, entertainment, virtual reality, and so many others. The evolution and execution and execution of drones set off essential regard for serviceability and accessibility. Unluckily as drones become common and their requirement increases, the drones are becoming progressively endangered for safety and privacy offensive, inclusive of, unlimited stuffing, exposure of secret and counterfeit. Towards managing these issues and safety warnings, a genuine blueprint of vigorous safety convention is needed [14]. However, much researches have been done on these issues but still, there are some research gaps to be filled, specifically drone-based secure transmission assist for ideal forward confidential and providing authentication. The paper presented the protocols used before for privacy concerns in drones significantly for military purposes. The security is to be maintained between drone-to-drone transmission and between drones to base station transmission. The protocol not only supports general security but also gives ideal and confidential authentication. This protocol is ideal for supporting military transmission and also plays a role in the attack, crackers, fraud and identity theft, and D2D security [12].

### A UAV-assisted blockchain-based communication

The remarkable development of cryptographic ledger technology in the department of the domestic system has designed a relevant effect and accordingly, the department of the military has a significant effect on it. In military circumstances, many equipment and smart gadgets are linked. Drones gathered with advanced computing can provide simultaneous assistance. Although, with the use of the drone, smart gadgets and expert robotics strikers can reliably retrieve. This conducts disturbance on functionality and discloses the details securely to rivals. To overcome the issue a cryptographic technology is proposed. This gives a solution to prevent hostile denial of service hacks and secure information dropping to guarantee the safety of D2D transmission in the military channel to give enhanced security [27].

Blockchain-Based Routing Beyond 5G [26] Privacy, security, and safety in autonomous vehicles are more important factors in which blockchain technology is one of the highlighted techniques. The most sensitive application is in the military domain. In the military, the primary factors are privacy, security, and latency which cannot be compromised and are collected under the internet of military vehicles (IoMVs). The privacy and security of sender, receiver, and data paths for IoMV are achieved, for this blockchain technology that is a blockchain-based onion routing protocol for B-IoMV for the above-mentioned factors improvement. Although the blockchain scheme is very expensive. To overcome this, we use Interplanetary File System (IFS) and make it cost-effective and achieve good and improved results.

### Military communications over 5G networks

5G systems are state of the art for experiments around the world and disposal will launch into preliminary 2020. This paper is conducted on the use of 5G systems for military purposes. Many 5G techniques like mm-wave, enormous MIMO, software-defined networking, network function virtualization, heterogeneous network, D2D transmission, and the provocations in the path of 5G are studied in the paper. Then this paper concentrates on safety issues and services stepped up by the 3GPP group. Upcoming in combat area 5G techniques are introduced and 5G deployment globally with distinctive referred 5G expansion attempts by the government, the issues and the occasions onwards [4].

### Covert communication

UAV and D2D transmission are collectively assumed to provide global connection and a speed that is high-rate for critical and provide support transmission of information ahead in the wireless network. Although conventional encoding scheme still not able to prevent information from the enemy and further based on the information from transmitter and receiver can easily attack. However, to make strong privacy of this information covert communication is the main requirement to hide the information. Firstly, D2D transmission was implemented in the UAV then find the problems of mode selection and cooperative jamming/interference in the communication. The aim is to provide a highly secure solution for sensitive information. For this, two D2D architectures in which each UAV performed as a flying base station or aerial user equipment. Then by the combination of mode selection and cooperative jamming we propose covert communication, the mode selection can switch to half-duplex or full-duplex modes and cooperative jamming means the interference is rejected by injecting D2D pairs to confuse enemies. The goal is to increase the channel rate and the probability to detect the enemy is very high [3].

### Comparison of D2D communication in military networks

In military networks, device-to-device (D2D) communication allows units to exchange data directly and with minimal delay without depending on centralized infrastructure. Faster decision-making, better coordination, and resilience in hostile or infrastructure-limited contexts are all made possible by D2D networks as opposed to conventional networks. Secure authentication, managing interference, and preserving connectivity while moving around are some of the difficulties it confronts, though. Military D2D is an essential part of contemporary tactical communication systems as, in contrast to civilian uses, it must priorities resilience, encryption, and adaptation to changing situations. Details are as follows:

## UAV-based D2D communication for social networking

Device-to-Device (D2D) communication via UAVs provides a dynamic and adaptable way to improve social networking, particularly in places with unstable infrastructure. Users can communicate directly with one another thanks to Unmanned Aerial Vehicles (UAVs), which can serve as mobile base stations or relays. In crowded areas or far-flung locations, this method guarantees dependable data transfer, increases coverage, and enhances connection. Because UAVs can communicate via line of sight, they can facilitate proximity-based services, live broadcasting, and content sharing. In social networking contexts, UAV-assisted D2D transfers data from conventional infrastructure to nimble, airborne communication nodes, improving user experience, facilitating real-time engagement, and lowering network congestion. Details of solution UAV based UAV-Based D2D Communication for Social Networking is described in the figure below;

This diagram demonstrates how UAVs can support social networking applications through D2D communication. UAVs facilitate peer-to-peer communication between devices in environments like crowded events or festivals, where traditional infrastructure might be overwhelmed, enabling real-time content sharing.

This figure presents a scenario in which D2D transmission is applied to social networking environments. UAVs serve as mobile access points, enabling direct communication between users, enhancing user experience and reducing reliance on traditional network infrastructures in high-density areas.

### UAV-supported social networks

The aggregate D2D transmission technique can upgrade the system functions for drones assist extensive social networking (ESN). The paper increases the sum social category advantage of the social system by energy minimization, while QoS demands all D2D end-users must be contented. In the presented technique, the physical obstruction and social linkages among end-users in the social territory, independently. Nevertheless, the issue is interdependent to overcome this we use an invisible non-convex element and estimate the real challenge with more controllable convex challenges. We present a method by divided convex problems into small sub-problems which are simple and give immobilized solutions to a real challenge [38].

### Social networks and D2D communications

In [22] recent years, the foremost feature of the latest technology evolution is the continuously growing requirement for information transfer between numerous kinds of gadgets, both variable and stationary. According to this the straightforward transmission among gadgets establish the latest, position-based P2P connection and services, and unload congestion in cellular systems. The important obstacle for this type of D2D transmission is productivity, bandwidth effectiveness, and jitter. Some of these obstacles are resolved by applying the social IoT model of equipment and human elaborate combined in the system network led without any control (autonomously) with the help of social connection by following the set of regulations.

#### Social network-aware D2D communication in wireless network

To offer new applications in the wireless domain D2D transmission is quite near to that wireless capacity. In this regard, the need is to optimize D2D a socially aware approach was proposed which worked on two layers included the social network layer and the physical wireless network layer. In physical layer network is captured by the user and a technique to optimize traffic offloading was also proposed in D2D transmission by obtaining contents from the online social networks. The data rates of the network increased [35].

### Cascading failure in D2D-based social networks

To avoid cascading failure in D2D SNS, increment in the number of links in a physical network the author proposed the placement of devices algorithm. To increase the link in a physical network the main solution is to increase the number of relay devices. In this matter, UAV should be able to for D2D transmission and the base station should also support D2D links. To increase relays, optimization was taken into account to reduce node resilience and then relays were placed on this optimization solution. In this way, the relay was placed without complete information of the physical network [32].

### Comparison of D2D communication in social networks

None.

## D2D communication in Ad-Hoc networks

In ad-hoc networks, UAV-based Device-to-Device (D2D) communication provides an adaptable and effective way to improve connection in dynamic, infrastructure-less contexts. When conventional infrastructure is inaccessible or broken, Unmanned Aerial Vehicles (UAVs) serve as mobile relays or access points that allow direct communication between ground equipment. This method enhances network scalability, coverage, and dependability in situations such as military operations, remote sensing, and disaster recovery. In order to minimize interference and preserve line-of-sight connectivity, UAVs can dynamically change their locations. In ad-hoc networks, its combination with D2D communication guarantees smooth connectivity amongst widely dispersed or mobile users while improving data throughput and lowering latency. UAV based solution of D2D transmission in ad-hoc networks are described in the figure below:

This diagram highlights the role of UAV-based D2D communication in ad-hoc networks. The visual focuses on scenarios where UAVs establish temporary communication links between devices in environments such as disaster zones or military operations, where no pre-existing infrastructure is available.

This scenario-based diagram illustrates D2D transmission over an ad-hoc network, showcasing UAVs as autonomous communication relays between devices that are not connected to fixed infrastructure. It emphasizes the dynamic nature of ad-hoc networks, where UAVs create temporary, flexible communication links [36].

### Vehicular Ad-Hoc networks for VANETs

The functions of the LTE technique have developed from ground base station-dependent disposal in authorized bands to the latest scenarios which make up for ad-hoc, D2D transmission, and unauthorized bands functions. Roadway transmissions like (vehicular) is an advancing department of specific for LTE make up for our comprehension for both (autonomous devices) cars and drone. The current domestic appliance is created for a ground base station which makes it undesirable for roadway equipment functions that demand lightweight and unauthorized band [7]. For this, we propose tiny-LTE a network created that gives fully autonomous, multitasking, and tight LTE cells by using open-source applications. Because of its small size, the lightweight tiny-LTE network authorizes mobile disposal on board vehicles and UAVs.

This authentic the optimality of the tiny-LTE proposal and minimize the range of transmission.

#### Cluster-based D2D-architecture for safety services in vehicular ad-hoc networks

It is critical and all-important for road traffic authorities to establish approaches to manage the existence of autonomous and legacy vehicles. To deliver a message in a time with efficiency and secure privacy becomes critical for traffic management in the future. Most of the work on the VANET vehicular ad-hoc network was carried out to overcome the challenge. However, from the researches advanced LTE-based systems may require the resources to provide support to vehicle-to-vehicle transmission. In the paper, an LTE-based cluster scheme was proposed for D2D architecture to achieve high rates of packet delivery for the secure vehicular ad-hoc network transmission [23].

### Flying Ad-Hoc networks (FANETs)

Drones formerly essential to military administrations are currently detecting their path in most of the domestic and civilian implementations. If the government allows and authorize drone to perform tasks autonomously then the skies are over-crowded with many tiny drones and all are doing different assignments like delivery and mail data packets, traffic management, entertainment, agriculture monitoring, weather forecasting, wildlife monitoring, and rescue operations. Thus, by the use of new technologies and control, all small UAVs with one large UAV to a single network are called flying ad-hoc networks. They are very flexible, easily disposable, and have minimal operating costs [24]. This provides a flexible transmission system between drones to a base station. They have other challenges as well important of which is transmission, broadcasting, bandwidth. These challenges are resolved by enhancing the flying ad-hoc capabilities.

### Delay-tolerant Ad-Hoc networks (DTNs)

Device-to-device transmission is a way of communication that has confirmed its importance in ad-hoc networks as an advanced directive. It allows all mobile gadgets to communicate on a licensed frequency with each other under cellular network control. The probability of D2D transmission is carried out in different and contrasting scenarios. To improve the probability of D2D transmission in ad-hoc networks the scheme of delay-tolerant networking (DTN) was introduced. DTN enables infrequent communication in ad-hoc networks [37].

### Comparison of D2D communication in Ad-Hoc networks

In ad-hoc networks, device-to-device (D2D) communication allows direct data exchange between adjacent devices without the need for centralized infrastructure. In contrast to traditional networks, D2D in ad-hoc setups offers more flexibility and faster data transmission, particularly in remote or infrastructure-less environments. Despite these drawbacks, such as dynamic topology changes, limited transmission range, and energy constraints, D2D is a highly suitable solution for temporary, emergency. Details are tabulated below:

## UAV-based D2D communication in IoT

In the Internet of Things, UAV-based Device-to-Device (D2D) communication presents a unique way to improve connection in isolated or infrastructure-constrained locations. IoT devices may communicate directly with one another thanks to drones (UAVs), which can act as mobile relays or data collectors. This technology improves coverage and efficiency in difficult-to-reach areas by reducing dependency on conventional network infrastructure. Data transfer may be improved by UAVs using D2D technology, which can independently create high-bandwidth, low-latency communication networks between components. In Internet of Things applications, this method guarantees smooth data transfer for environmental sensing, disaster recovery, and real-time monitoring, allowing for more dependable and effective communication in a variety of situations, including emergency response, surveillance, and agriculture. UAV based D2D transmission in IoT solutions networks are described in the figure below;

This diagram demonstrates D2D transmission within IoT-based networks, showing how UAVs facilitate communication between IoT devices in environments like smart cities or industrial IoT applications. UAVs enable low-latency and high-throughput communication, ensuring efficient data exchange between devices.

This figure presents a real-world scenario where UAVs support IoT-based D2D communication. The diagram illustrates how UAVs connect IoT devices to each other in a network where traditional infrastructure might not be available, improving scalability and network resilience in IoT applications.

### Emergency communication in IoT

The IoT has remarkable significance far away fifth causation transmission networks. Although, the IoT is at risk of catastrophe due to the network being deep energy and the gadgets being exquisite. In this paper, a drone-guided transmission in extremity scenarios and diverse IoT and NOMA technique is presented which does not need any obstruction cancellation. To help the transmission of the sustainable end-users and IoT gadgets effectively. A multipurpose resource issuing technique is presented for drone-guided heterogeneous networks. Firstly, the challenge is decoupled by initializing power. Then by using another algorithm the gadgets are divided into sub-channels. Lastly, the technique is used to match the power transmitted between end-users and drones. The proposed method yields a desirable functionality of the drone [21].

### UAV IoT coverage in disasters

When natural catastrophe attacks, the broadcasting for IoT is intensively damaged because of the destroyed transmission with a ground base station. Drones may utilize a flying central station to give on-the-spot broadcasting for IoT because of its mobility and reliability. In this paper, an antenna array system is created and multi-hop D2D transmission to permit flexible communication and enhance the drone broadcasting for IoT in catastrophe. Firstly, a D2D connection is developed to enhance the broadcasting of drones because of constrained power. A short path algorithm is designed to avoid obstruction. The transceiver is designed to increase the functionality and performance of drone transmission [[12], [34]].

### Wireless powered public safety IoT

The use of transmission inpublic safety scenarios with the help of IoT devices to provide connection in disaster-affected areas. The UAVs support wireless networks for the improvement of energy in terms of efficiency in a way using the non-orthogonal multiple access (NOMA) techniques. The IoT devices form affiliation firstly by choosing their part in the network and in the distributed manner. The members indulge in stochastic learning to associate with affiliation heads using a reinforcement learning scheme. To increase the lifetime of public safety IoT devices harvest energy from the mobile UAV before transmission of any kind of information. The optimization technique is used for the correct positive of UAV placement in 3D and nodes switch in emergency situations play a very important role [[6], [25]].

### Secure D2D communication for 5G IoT network

D2D transmission over 4G and 5G networks were discussed. The advantage of 5G over 4G is that it provides the reliability of communication from vehicle to every gadget, which highly supports the autonomous operation. However, 4G faced security issues including free riding attack and privacy sniffing. In the paper, the IoT was discovered along with 5G to give ultra-reliable low latency in which then security issues become more complex due to the resource-constrained behavior of IoT [5]. To overcome the challenge of security in IoT devices in a 5G network a lightweight cryptography scheme and a D2D transmission were used to provide secure authentication and keep the data confidential. A survey was done in the domain to give security and limitations [31,47].

### Comparison of D2D communication in IoT networks

In Internet of Things networks, device-to-device (D2D) communication allows devices to exchange data directly without going via centralized infrastructure, increasing speed and decreasing latency. D2D in IoT offers reduced communication overhead, enhanced scalability, and superior energy efficiency as compared to conventional techniques. However, issues like security, mobility management, and interference still exist. D2D communication is useful when used in fields like smart homes, healthcare and industrial IoT, but it requires strong protocols to guarantee dependable and safe data transfer. Details are tabulated below:

## Conclusion

D2D transmission in this paper over different networks is addressed in this paper. The complete overview of UAV-based D2D transmission, opportunities, challenges, and beyond 5G environment discussed. D2D over cellular, 5G, in public safety, military, social, ad-hoc, and IoT-based transmission described comprehensively. Reliable transmission, distributed resources, power allocation, performance analysis, and interference mitigation in cellular networks are discussed. In a 5G environment the management of interference, security protocols, routing algorithms, relay selection, challenges, issues, and solutions are described. Multi-hopping, connectivity, clustering, and MIMO relaying for national security and public safety based on UAV mentioned in this paper. Protocols and future sensitive applications, blockchain technology, jamming, interference, mode selection, covert communication, 5G environment, and internet of military vehicles in the military domain are introduced. Autonomous relaying, device placement, and wireless networks over social networks are also discussed. Over ad-hoc networks, the concept of clustering in VANETs and lightweight ad-hoc deployable cellular networks and the challenges are presented in the paper. NOMA, lightweight cryptography, IoT coverage in disaster, multi hopping, and an array of antenna designs are also presented under the IoT domain. This paper is a complete package of D2D transmission and future challenges are also given to provide a path in the same domain.

## Future directions

As UAV-based D2D communication continues to evolve, several areas require further research to enhance its capabilities within heterogeneous networks. A primary focus should be the integration of UAVs with 6G networks, where UAVs can significantly extend coverage and improve reliability in urban and remote areas. Future work should explore the use of terahertz communication and non-orthogonal multiple access (NOMA) in UAV-based D2D networks for 6G.

Another critical area is the autonomous management of UAV fleets. With the growing use of UAVs in various applications, AI-driven algorithms and machine learning models will be essential for optimizing coordination, flight path planning, and resource allocation.

Security remains a significant concern. Research should focus on lightweight cryptographic solutions and blockchain technologies to ensure secure communication, particularly in sensitive environments like military and critical infrastructure.

Furthermore, energy efficiency in UAVs is crucial for long-duration missions. Developing energy-efficient algorithms and exploring energy harvesting technologies will enhance the sustainability of UAV-based communication systems.

Finally, the deployment of hybrid UAV networks combining UAVs, 5G/6G, and satellite systems will offer improved connectivity, particularly in underserved areas, enabling autonomous air mobility (UAM) and large-scale UAV networks.

## Ethics statements

[Our work did not copied / involved data collection from social media platform].

## CRediT author statement

**[Amjad Ali and Aqsa Zehraa** Conceptualization, Methodology, Software, Validity Tests, Supervision and Data Curation. **Muhammad Nafees** Writing- Original Draft preparation, Reviewing, Editing and Validation. **Muhammad Awais Amin** Visualization and Validation]

Supplementary material *and/or* additional information [OPTIONAL]

“None”.

## Declaration of competing interest

The authors declare that they have no known competing financial interests or personal relationships that could have appeared to influence the work reported in this paper.
